# iTRAQ-based Protein Profiling and Fruit Quality Changes at Different Development Stages of Oriental Melon

**DOI:** 10.1186/s12870-017-0977-7

**Published:** 2017-01-28

**Authors:** Xiaoou Guo, Jingjing Xu, Xiaohui Cui, Hao Chen, Hongyan Qi

**Affiliations:** College of Horticulture, Key Laboratory of Protected Horticulture of Education Ministry and Liaoning Province, Collaborative innovation center of protected vegetable suround Bohai gulf region Shenyang, Shenyang Agricultural University, Liaoning, 110866 People’s Republic of China

**Keywords:** iTRAQ, Development Stages, Proteomics, Oriental Melon, Gene Expression

## Abstract

**Background:**

Oriental melon is one of the most popular crops for its nutritional and flavour quality. Components that determine melon quality, such as sugar, colour, texture, flavour and aroma, among other factors, accumulate in different developmental stages. Thus, correlating the proteomic profiles with the biochemical and physiological changes occurring in the oriental melon is very important for advancing our understanding of oriental melon quality in the ripening processes.

**Results:**

iTRAQ-based protein profiling was conducted on ‘YuMeiren’ oriental melon fruit at different developmental stages. Physiological quality indices, including firmness, rind colour, soluble solids content (SSC), ethylene production, sugar content and volatile compounds were also characterized during four maturity periods of the melon, including 5, 15, 25 and 35 days after anthesis (DAA). A principal component analysis (PCA) revealed that the aroma volatiles at 5 DAA and 15 DAA were similar and separated from that of 35 DAA. More than 5835 proteins were identified and quantified in the two biological repeats and divided into 4 clusters by hierarchical cluster analysis. A functional analysis was performed using Blast2GO software based on the enrichment of a GO analysis for biological process, molecular function and cellular components. The main KEGG pathways, such as glycolysis, α-linolenic acid and starch and sucrose metabolism, were analyzed.

The gene family members corresponding to differentially expressed proteins, including lipoxygenase (*CmLOX01-18*) and alcohol acetyltransferase (*CmAAT1-4*) involved in the α-linolenic acid metabolic pathway, were verified with real-time qPCR. The results showed that the expression patterns of 64.7% of the genes were consistent with the expression patterns of the corresponding proteins.

**Conclusions:**

This study combined the variation of the quality index and differentially expressed proteins of oriental melon at different developmental stages that laid the foundation for the subsequent protein and gene function validation.

**Electronic supplementary material:**

The online version of this article (doi:10.1186/s12870-017-0977-7) contains supplementary material, which is available to authorized users.

## Background

Oriental melon (*Cucumis melo* var*. makuwa* Makino) is a species of thin-pericarp melon, and it has a sweet and crisp taste, juicy flesh, intense volatile aromas compound and the largest plantation in china [1]. During oriental melon ripening, components of the quality index, such as sugar, colour, texture, flavour and volatiles etc., change significantly [2]. At the same time, a large number of proteins and genes expression changed concomitant with ripening [3–8].

Proteomics is the large-scale study of proteins, especially their structures and functions [9]. This approach can systematically explore the physiological and biochemical changes of plants and dynamically describe the differences in the expression levels of different proteins [10–14]. There are many proteomics research methods, such as two-dimensional gel electrophoresis (2-DE), difference gel electrophoresis (DIGE) and isobaric tags for relative and absolute quantification (iTRAQ) [15]. The iTRAQ labelling technique is a quantitative technology for proteomics that uses 4 or 8 kinds of isobaric tags developed by American ABI (Applied Biosystems Inc.) in 2004. By using two-dimensional liquid chromatography tandem mass spectroscopy, this labelling technique can be used to make relative and absolute quantification analyses for up to eight samples simultaneously [8, 16–18].

In recent years, the application of proteomics to research studies on fruit ripening has progressed in climacteric and non-climacteric fruits [19–21]. Strawberries are a non-climacteric fruit, and it was found that more than 892 proteins are involved in the metabolic pathways of strawberries, including flavonoid/anthocyanin biosynthesis, volatile biosynthesis and allergen formation [22]. More than 630 proteins were identified and quantified in the grape berry during ripening, and these proteins were involved in photosynthesis, carbohydrate and malate metabolism, and other pathways. Other non-climacteric fruits also have differential changes in proteins, such as citrus, cherry and honey pomelo [23, 24].

In climacteric fruits, 988 protein spots were identified in apples during ripening and were involved in the lipoxygenase (LOX) pathway for synthesizing aromatic substances as well as in the synthesis and degradation of starch [25]. Two varieties of peach fruit had differential protein expression for 53 proteins before and after climacteric conditions, and these proteins were related to basic metabolism, secondary metabolism, ethylene synthesis and the stress response [26]. The waxberry had 43 differentially expressed proteins at different developmental periods that were mainly related to the metabolism of sugar and energy, anthocyanin metabolism and the stress response as well as defence and other properties [27]. The oriental melon is a typical climacteric fruit; however there are limited reports on the proteomics for oriental melons at different ripening stages.

In this study, we analysed physical and chemical properties, such as firmness, rind colour, soluble solids content (SSC), soluble sugar content, ethylene content and aroma volatiles in four maturity periods of melons, including 5, 15, 25, and 35 days after anthesis (DAA). We used iTRAQ to research and identify the differentially expressed proteins in melon fruit ripening. Expression analysis was conducted for genes (e.g., *CmLOX01-18* and *CmAAT1-4*) related to substances participating in α-linolenic acid metabolism to further verify the expression patterns of corresponding proteins.

## Methods

### Plant materials

Oriental melons (*Cucumis melo* var. *makuwa* Makino) cultivar ‘YuMeiren’, from the Yijianpu Mishijie Melon Research Institution, Changchun,China, were individually grown in pots (volume of 25 L with a soil:peat:compost ratio of 1:1:1) in a greenhouse at Shenyang Agricultural University, Shenyang, China, from March through July 2015. Spacing in the rows was 60 cm, and the distance between rows was 80 cm. Female flowers were pollinated with ‘Fengchanji2’ (a hormone complex, which mainly contains 4-chlorophenoxyacetic acid to increase the rate of fruit set; Shenyang Agricultural University) to increase the rate of fruit set and tagged on the day of bloom. Melons were cultivated as single stems with 2 or 3 fruits per vine. Melons were harvested on 5, 15, 25, and 35 DAA. The physiological maturity of this oriental melon was approximately 35 days after anthesis (DAA). On average, a minimum of 30 fruit per stage were harvested for determining firmness, soluble solids content (SSC), rind colour, ethylene production, sugar content and volatile compounds. Two independent biological replicates with 30 pooled fruits each were used to conduct the proteomic analysis at each sampling time. For the proteomic analysis, the fruits were peeled, cut into small pieces, immediately frozen in liquid nitrogen and stored at −80 °C until further use.

### Firmness, rind colour and soluble solids content (SSC)

A fruit firmness tester (FHM-1, Takemura, Japan) calibrated with a 1 kg weight and equipped with a 12 mm diameter probe was used according to the methods of Mendoza [28]. Seven readings were obtained for each fruit at 2 pared surfaces on the equator and recorded in units of N/cm^2^ after a controlled deformation. A CR- 400/410 colorimeter (Konica Minolta, Japan) was used to detect the rind colour of melons. Six readings were collected from the equatorial zone of each fruit. Among these values, L* represents the brightness of the rind, which directly correlated with the fruit lustre. The label a* represents the red/green ratio, and higher positive values indicate red fruit, whereas negative values indicate green fruit. The label b* represents the yellow/blue ratio with higher positive values indicating yellow fruit and negative values indicating blue fruit. The SSC of fresh melon was measured on each melon by dropping the extracted juice from the equatorial region of flesh tissue onto a digital refractometer (DBR45, Huixia, Fujian, China) as described by Liu [29]. The firmness, rind colour and SSC experiments were performed in triplicate.

### Soluble sugar content

Fresh melon tissue, 1 g fruit weight (FW), was extensively ground and extracted 3 times in 5 mL 80% (v/v) ethanol at 80 °C for 1 h. After extraction, the extracted liquid was mixed and evaporated to dryness in an evaporating dish over an 80 °C water bath. The residues were re-dissolved in 1 mL ultrapure water and passed through 0.45 μM filters. Then, a 5 μL sample was injected into an HPLC (Waters 600E) equipped with a carbohydrate column and an Alltech 2000ES evaporative light detector. A mixture of 80% acetonitrile with 20% ultrapure water (v/v) was used as the mobile phase, and the flow rate was set at 1 mL min^−1^. Fructose, glucose and sucrose were identified and quantified from the retention times and peak heights of sugar standards. Each measurement was repeated three times.

### Ethylene content

The ethylene in the melon cavity was extracted and determined with a Varian GC-3800 gas chromatograph three times. Briefly, 50 μL of a 1 mL gas sample was injected manually by using a micro-syringe (Shanghai Gaoge Industrial and Trading Co., Ltd) into a Varian (GC-3800) equipped with a flame ionization detector (FID) and fitted with a chromatographic column (GDX-102, 3 m × 2 mm i.d., Dalian Institute of Chemical Physics, China). Analyses were run isothermally with an oven temperature of 100 °C, a split/splitless inlet system with a 1041 injector held in splitless mode at 250 °C, and a detector temperature of 120 °C. The injector insert for 0.53 mm i.d. columns was stainless steel (Part No. 392543101, Varian). The samples were separated into a 30 m × 0.32 mm i.d. × 4 μm thickness capillary column (CP8567, CP-silica PLOT, Varian) in splitless mode and maintained at 100 °C. Nitrogen was used as the carrier gas. The flow rates for nitrogen, hydrogen and compressed air were 20, 30 and 300 mL min^−1^, respectively. Ethylene was quantified by the peak area, and the external standards were used for calibration. The calibration curve was linear when the concentration of ethylene was in the range of 10 to 50%, v/v (μL/L) (*r* = 0.997) [30]. Each experiment was performed in triplicate.

### Volatile analysis

The volatile compounds of different stages of melon ripening were detected with headspace (HP)-solid-phase-micro-extraction (SPME)-gas-chromatography-massspectrometry (GC-MS), as described by Liu and Tang [29].

Frozen melon samples (100 g of flesh) were thawed at room temperature for 30 min. Fresh juice was squeezed with a juicer (JYL-C05, China), and juice samples were collected by filtering juice through a glass funnel and four layers of cheesecloth. Then, 3.5 g sodium chloride (analytical grade) and an internal standard (50 L of 1-octanol, 59.5 mg L^−1^, 0.5%, v/v, Aladdin Chemistry, China) were added to 10 mL of juice supernatant. The mixture was homogenized completely and poured into a 20 mL glass vial (Thermo, USA). The vials were sealed using a crimp-top cap with silicone/aluminium septa seals (20 mm, Thermo) and heated at 40 °C in a water bath. Then aroma volatiles were extracted from the headspace for 30 min with a SPME fibre (100 m polydimethylsiloxane) with 1 cm long standard needle for manual operation (Supelco, 57347-U, Bellefonte, PA, USA), which was previously preconditioned at 250 °C for 30 min in the gas chromatography injection port. The SPME needle was from Supelco (57347-U, Bellefonte, PA, USA) and the GC-MS was from Thermo Scientific (TraceGCUltra-ITQ900, Waltham MA 02454). The GC system was equipped with a 30 m∗0.25 mm∗0.25 μm thickness capillary column (ThermoTR-5msSQC, USA). Each experiment was performed in triplicate.

### Protein sample extraction

Acetone precipitation method was adopted to extract protein from 2 g sample. Took out 2 g sample and put it into 5 mL EP tube. Two stainless steel beads were put into each sample tube. Added 2 mL SDT dissolution buffer (4% SDS, 100mM Tris–HCl, 1mM DTT, pH7.6); 60 rpm, 5 min, beads beating, breaking up the tissues in flesh by oscillator; 100 W, 5 min, ultrasonic decomposing; incubating for 5 min at 95 °C, reductive cleavage; 15000 g, 15 min, extract the supernatant by centrifugation for 2 times; the supernatant was added with 7 times volume acetone precipitated protein and incubated overnight, 15000 g*4 °C, centrifuging for 20 min to remove the supernatant; added with 1 mL acetone, crushed and precipitated; after being placed for 30 min at −20 °C, 20000 g*4 °C, centrifuging for 15 min to remove the supernatant; air drying the remained acetone in precipitation, added with appropriate SDT, washed by ultrasonic wave for 5 min, incubated for 5 min at 95 °C; 15000 g centrifuging for 15 min, took out the supernatant to measure the quantity. The concentration of sample was detected by fluorescence spectrophotometry based on tryptophan concentration. The integrity of sample was detected by polyacrylamide gel electrophoresis [31–33].

### iTRAQ labeling

For each developmental stage, a volume corresponding to 50 μg of protein was precipitated with 20 volumes of acetone at −20°C overnight. After centrifugation for 10 min at 15300 × g, the protein pellet was dissolved in 20 μL of iTRAQ dissolution buffer (Applied Biosystems) containing 2% (w/v) SDS. Proteins were reduced and alkylated in 3 mM tris-(2-carboxyethyl) phosphine (TCEP) and were incubated for 1 h at 60 °C. The peptides were labeled using iTRAQ 8-plex kits (Applied Biosystems, USA) according to the manufacturer’s protocol. Peptides of oriental melons at different ripened stages (5, 15, 25, 35 days) after anthesis were labeled with iTRAQ tags 113, 114, 115, 116, 117, 118, 119 and 121, respectively.

Since the sample solution contained the following several kinds of reagents: dissolution buffer, 75% organic solvent (ethyl alcohol and acetonitrile), 1 mM reducing agent (TCEP), 0.02% SDS, 5mM calcium chloride, excessive iTRAQ reagent, and these components might affect the subsequent mass spectrometry, so the sample must be purified by ion exchange chromatography before liquid mass spectrometric analysis [34–36].

### SCX fractionation

SCX chromatography was performed with a LC-20AB HPLC pump system (Shimadzu, Kyoto, Japan). The iTRAQ-labeled peptide mixtures were reconstituted with 4 mL of buffer A (10 mM KH_2_PO_4_, 25%ACN, pH3.0) and loaded onto a 4.6 × 250 mm Ultremex SCX column containing 5 μM particles (Phenomenex). The peptides were eluted at a flow rate of 1 mL min^−1^ with a gradient of buffer A for 10 min, 5–60% buffer B (10 mM KH_2_PO_4_, 1 mol L^−1^ KCl, 25%ACN, pH3.0) for 27 min and 60–100% buffer B for 1 min. The system was maintained at 100% buffer B for 1 min before equilibrating with buffer A for 10 min prior to the next injection. Elution was monitored by measuring the absorbance at 214 nm, and fractions were collected every 1 min. The eluted peptides were pooled into 20 fractions, desalted with a Strata X C18 column (Phenomenex, CA, USA) and vacuum dried.

### LC–MS/MS analysis based on Q Exactive

The peptides were dissolved in 0.1% FA and 2% ACN, and then centrifuged at 13500 *g* for 20 min. The LC–MS/MS was carried out using a Q Exactive MS (Thermo Scientific). The Q Exactive was interfaced with an UltiMate 3000 RSLCnano system. The peptide mixture was loaded onto a PepMap C18 trapping column (100 μm i.d., 10 cm long, 3 μm resin from Michrom Bioresources, Auburn, CA, USA) and then separated on the PepMap C18 RP column (2 μm, 75 μm × 150 mm, 100 A) at a flow rate of 300 nL min^−1^. Peptides were eluted from the HPLC column by the application of a linear gradient from 4% buffer B (0.1% FA, 80% ACN) to 50% buffer B for 40 min, followed by ramping up to 90% buffer B in 5 min. The eluted peptides were detected by Q Exactive and MS data were acquired using a data-dependent top20 method, dynamically choosing the most abundant precursor ions from the survey scan (350–1800 m/z) for HCD (high-energy collisional dissociation) fragmentation. Determination of the target value was based on Automatic Gain Control (AGC). Survey scans were acquired at a resolution of 70,000 at m/z 200, and resolution for HCD spectra was set to 17500 at m/z 200. Normalized collision energy was 30 eV and the under-fill ratio, which specifies the minimum percentage of the target value likely to be reached at maximum-fill time, was defined as 0.1%. The instrument was run with the peptide recognition mode enabled.

### Protein identification and quantification

Protein identification and quantification were performed with ProteinPilot™ Software 4.5 (AB SCIEX, USA) against the Cucumis melo.fasta (http://www.ncbi.nlm.nih.gov/protein/) using the Paragon algorithm. The utilized search parameters used were as follows:(1) Fixed modifications: Carbamidomethyl (C); (2) Variable modifications: Oxidation (M),Acetyl (Protein N-term); (3) Digestion: Trypsin; (4) Instrument: Triple TOF5600. For iTRAQ quantification, the peptide for quantification was automatically selected by the Pro Group algorithm to calculate the reporter peak area, error factor (EF) and the *p* value. The peptides and corresponding relative abundances were obtained in ProteinPilot using a confidence cutoff of >1.0 (>90%) and >1.3 (>95%) or the experiments of the green and ripe stages, respectively. Only the proteins identified with at least 2 different peptides and *p* < 0.05, and quantified with a ratio of >1.5 and *p* < 0.05, were considered to be differentially expressed proteins (FDR < 1%). The final fold change was calculated as the average value obtained from two replicates.

### Bioinformatics analysis

Functional analysis of the identified proteins was conducted using Blast2GO Software [37]. Hierarchical clustering analysis was conducted using PermutMatrix 1.9.4 software. Pearson distance and McQuitty’s algorithm were used for data aggregation.

### Real-time qPCR analysis

The total RNA was isolated with TRIzol Reagent (Takara, Japan). DNase I (Promega, USA) was used to remove genomic DNA. The total RNA extracted from fruit was used to generate cDNA samples via random priming with Superscript III reverse transcriptase (Invitrogen, Thermo Fisher Scientific, USA).

The cDNA samples were used as templates and were mixed with 10 μM of each primer and SYBR Green PCR Real Master Mix (Tiangen, Beijing, China) for real-time PCR analysis using the ABI 7500 Real Time PCR System and Software 7500 ver. 2.0.3 (Applied Biosystems, USA) as described in the manufacturer’s instructions. The temperature procedure was: 95 °C for 15 min; and 40 cycles of 95 °C for 30 s, 57 °C for 30 s, and 68 °C for 1 min. The fluorescence signal was collected during the elongation at 68 °C of every cycle. The oriental melon 18S rRNA was used as an internal control to normalize small differences in the template amounts. The LOX/18SrRNA, AAT/18SrRNA, SS/18SrRNA and SPS/18SrRNA ratios for all samples were related to the ratio for 5 DAA, which was set to 1. The primers used for real-time qPCR are listed in Additional file 1.

## Results

### Firmness, rind colour and soluble solids content (SSC)

**Fig. 1:**
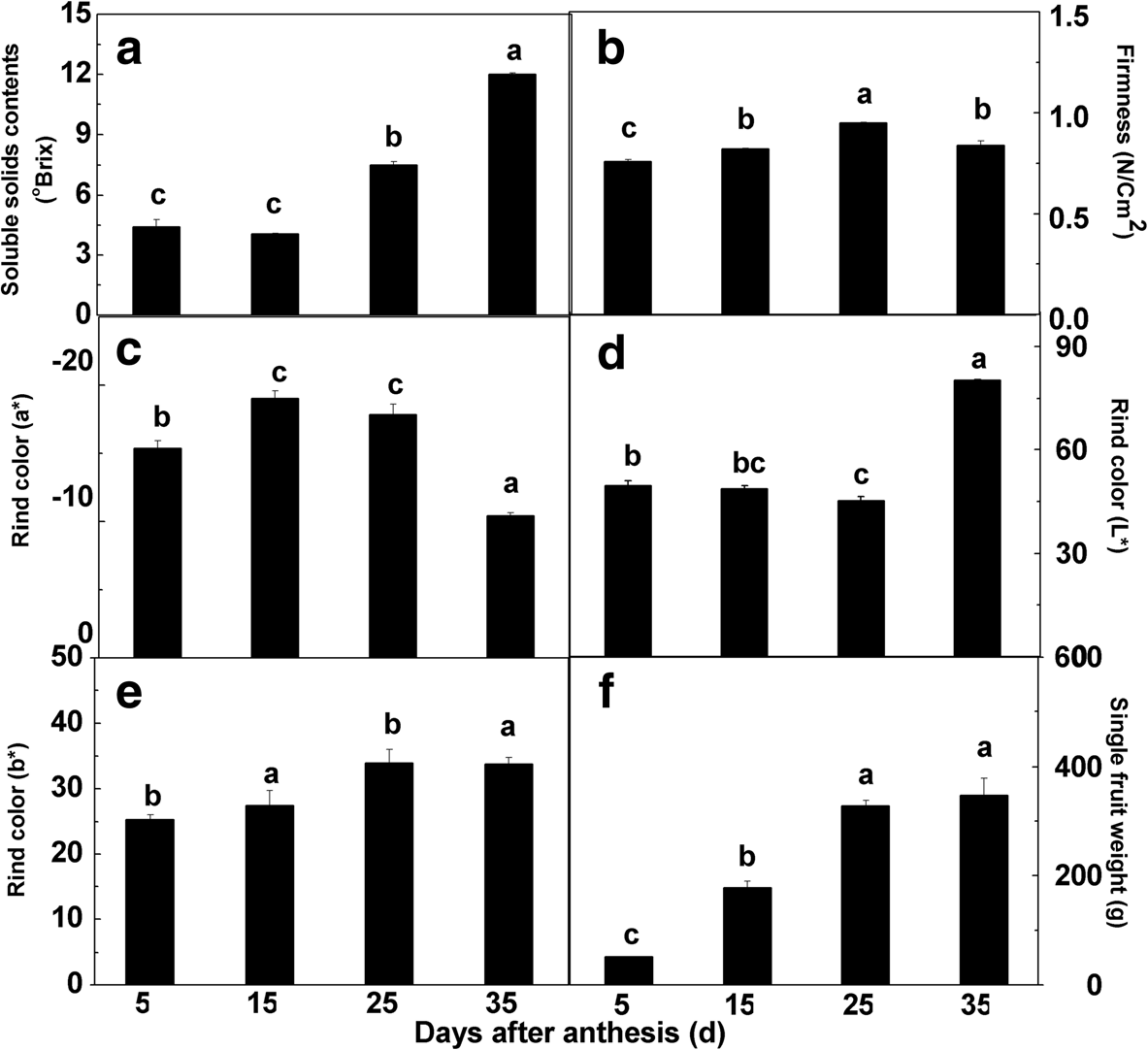
Physical sigins on different maturity periods of melon. **a** Soluble solids content, **b** firmness, **c**, **d** and **e** pericarp color. **f** per fruit weight. The four maturity periods of melon included 5DAA, 15DAA, 25DAA and 35DAA

The SSC in oriental melon increased and reached its maximum on 35 DAA (12.0%) in the whole process of growth and development (Fig. 1 ‘A’). The firmness initially increased, then reached its highest level at 25 DAA, and finally declined significantly (Fig. 1 ‘B’). The single fruit weight significantly increased during ripening (Fig. 1 ‘F’).

In the whole development period, the a*-value, b*-value and L*-value gradually increased (Fig. 1 ‘C’ ‘d’ ‘e’). This increase indicated that the fruit peel kept green in the early stages and then began to turn yellow, and it also showed that the brightness of skin tended to increase during maturation.

### Volatile compounds, soluble sugar content and ethylene content

**Fig. 2:**
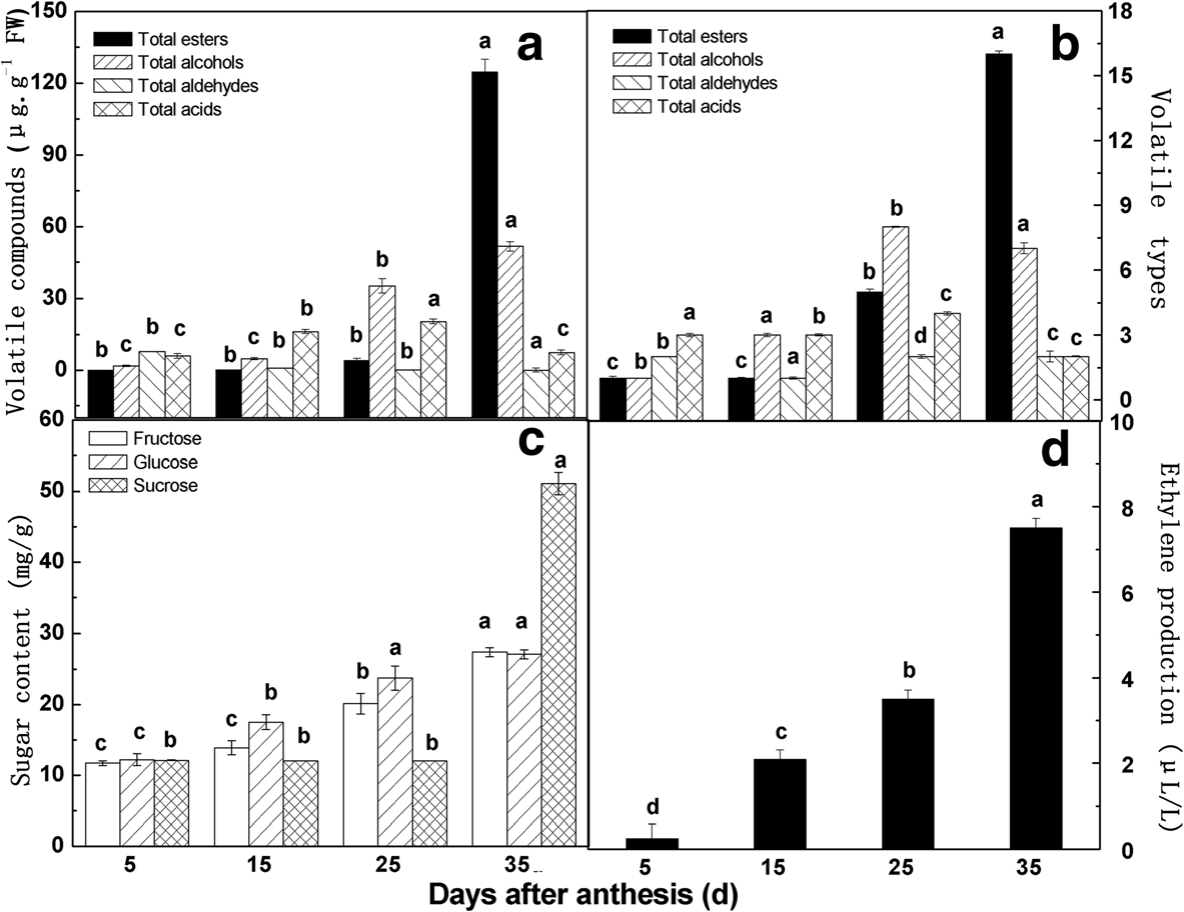
Volatile compounds, ethylene production and sugar content on different maturity periods of melon. **a** Volatile compounds **b** Volatile types **c** Suger content **d** Ethylene production. The four maturity periods of melon included 5DAA, 15DAA, 25DAA and 35DAA

The aroma volatiles in melon mainly include esters, alcohols, aldehydes and acids [38]. We identified a total of 40 volatile compounds in oriental melon during ripening (Additional file 2), including 17 esters, 12 alcohols, 4 aldehydes and 4 acids. The species and content of the 4 substance types remained stable at first, and then the alcohols and acids increased significantly at 25 DAA (Fig. 2 ‘A’ ‘B’). The species and content of esters increased significantly and reached their highest levels at 35 DAA, which indicated that the esters were the main aromatic determinants in melon tissues during the ripening period.

**Fig. 3:**
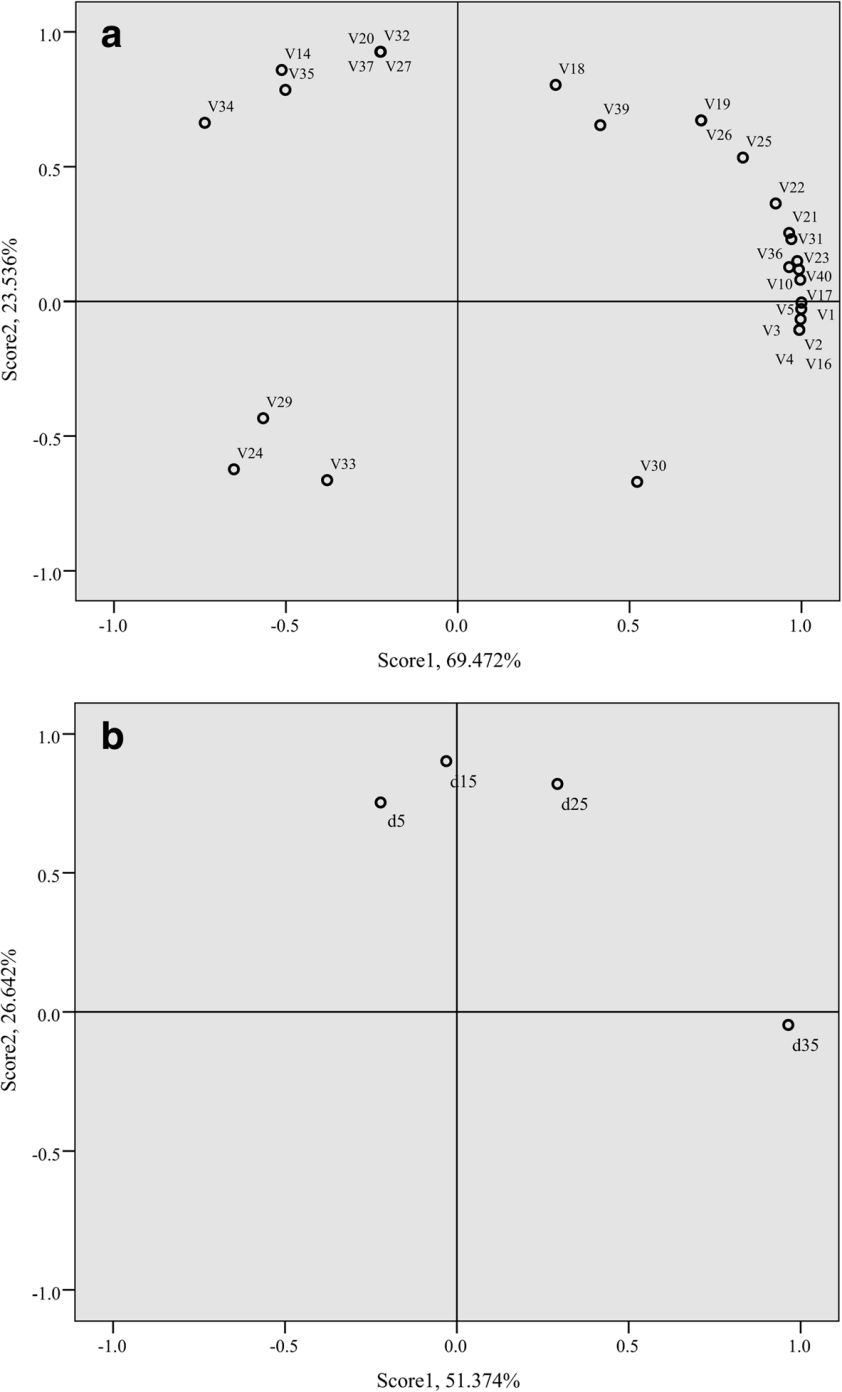
PCA of the aroma volatiles identified at different mature period of melon. The four stages of melon included “d5” (5DAA), “d15” (15DAA), “d25” (25DAA) and “d35” (35DAA). **a** Scores plots of the two main PCA of the aroma volatiles identified at the different mature period of melon. **b** Loading plots of the two main PCA of the aroma volatiles identified at the different mature period of melon. Codes were corresponding to the volatile compounds number in Additional file 2

The content of the aromatic substance was used as a variable in the principal component analysis (PCA). The first two principal components accounted for 93.008% of the total variability (Fig. 3 ‘a’). V1-V4 were esters and principal contributors to PC1. They separated from V34 and V35, which were acids. V20, V27, V32 and V37 contributed the most to PC2, and they were mainly alcohols and aldehydes.

The fruit at different development stages was used as another variable in the PCA (Fig. 3 ‘b’). The first two principal components accounted for 93.008% of the total variability. The 5 DAA measurements were similar to the 15 DAA measurements, and both were separated from the 35 DAA, which was principal contributors to PC1.

The inherent quality of melon fruit was closely related to the accumulation and composition ratio of sugar. The melon fruit mainly contained glucose, fructose and sucrose. Of the three soluble sugars, fructose and glucose accumulated slowly and synchronously. Sucrose rapidly accumulated and reached its maximum at 35 DAA (Fig. 2 ‘C’), which indicated that in the late stages of development, the accumulation of sugar accelerated. Fig. 2 ‘D’ shows that the ethylene content increased significantly during ripening and reached its maximum value at 35 DAA.

### Identification and quantitative results of differential proteins

**Fig. 4:**
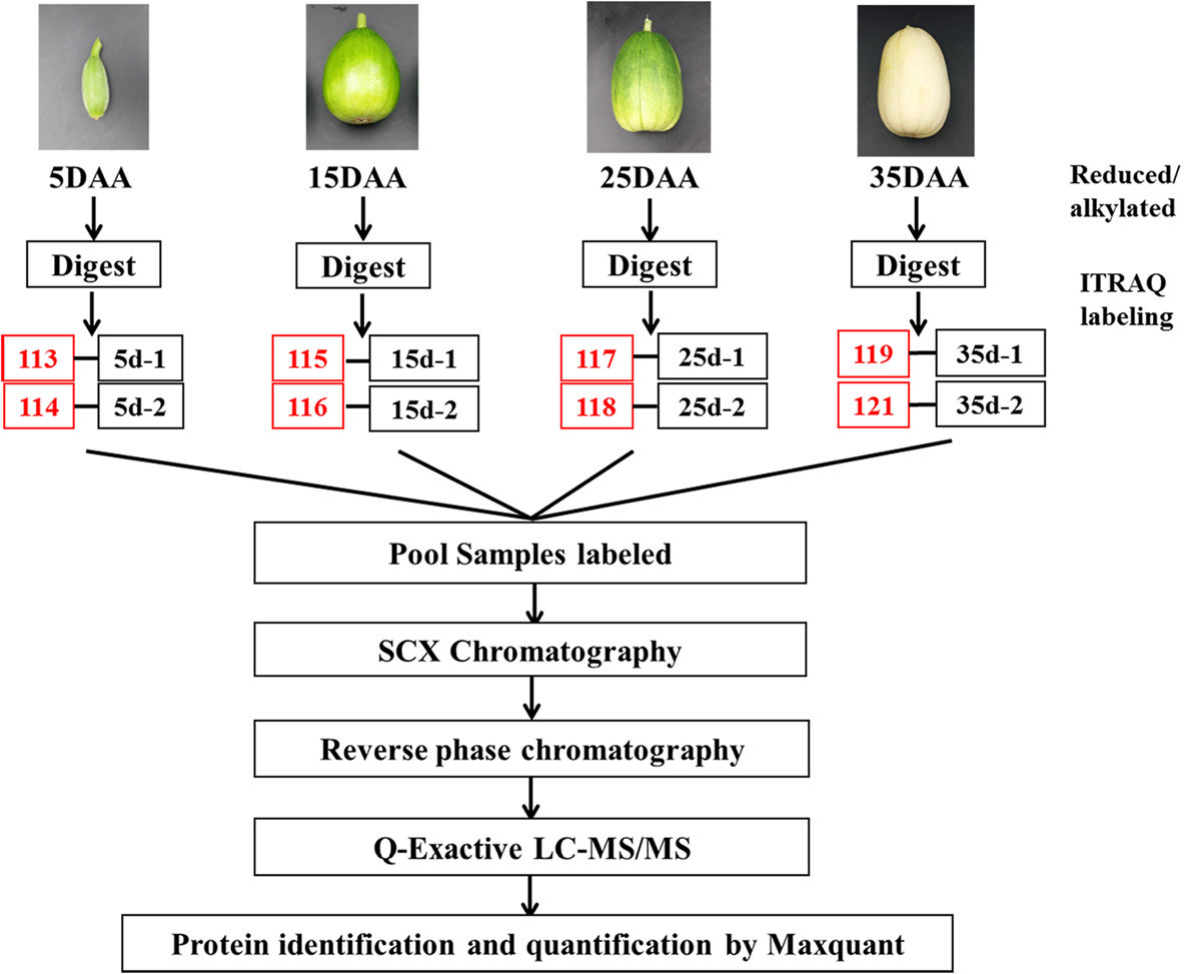
General workflow of the melon fruit ripening study employing proteomic technique of iTRAQ

Figure 4 describes the experimental design and workflow. In Additional file 3, the protein identification list showed that 5835 proteins were identified in this experiment, the total amount of peptides was 65426 and the amount of unique peptides was 36297. The evaluation for identification and quantitative results revealed that the scores for the peptides were satisfactory. Approximately 90% of peptides scored over 30 points (Additional file 4). The relative molecular mass of most proteins was between 0 and 100 (Additional file 5). The number of amino acids in the peptide sequence was between 5 and 15 (Additional file 6). The number of identified peptides corresponding to a type of protein was between 0 and 20 (Additional file 7). A relatively good correlation between two different proteins was found (Additional file 8).

**Fig. 5:**
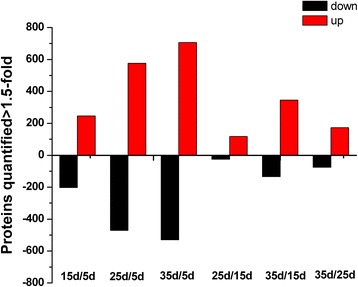
Overall changes in the protein level throughout melon development. Each value represents the number of sequences quantified between two developmental stages, for all the proteins that were up- (*red bars*) and down-regulated (*black bars*). An arbitrary fold change cut off of ± 1.5 was used to select the protein subsets

All proteins in these four stages were compared in pairs. Additional file 9 shows the results for proteins that were differentially expressed in the four maturity stages with two replicates for each measurement. Figure 5 illustrates the number of up- and down-regulated proteins between two stages. Of the differentially expressed proteins, three classes of developmental stages can be formed: stages with very few changes (25d/15d and 35d/25d); stages with many changes (25d/5d and 35d/5d); and stages with an intermediate number of changes (15d/5d and 35d/15d). Among these stages, 35d/5d was the developmental stage in which the largest number of proteins changed. These results indicated that the identification and quantification of these differentially expressed proteins revealed a change in protein abundance that was related to fruit maturation, and the most important changes at the protein level occurred in a non-adjacent maturation period for oriental melon.

### Clustering analysis of differential proteins

The clustering analysis is a common exploratory data analysis method. The goal is to group and sort data based on similarity [22]. In the results for clustering and grouping all differential proteins, the similarity of data patterns of the same samples repeated two times was higher, while the data pattern between different samples was lower (Additional file 10). This outcome further indicated that in the developmental process of oriental melons, the profile of proteins was different during ripening and the test repeatability of samples in the same stage was reliable.

To further explore the profile of differentially expressed proteins in different developmental stages, we conducted a timing analysis for 1694 differentially expressed proteins. First, data were pre-processed with the following steps: for each differential protein, the values of two biological replicates at the same time point were averaged; the logarithm of expression quantity acquired above base 1.5 was calculated to make the expression quantity of all proteins approximate the normal distribution; and for particular proteins, the average of expression quantities for 4 time points was calculated and then the datum for each time point was divided by the average expression quantity and subtracted by 1. The relative expression quantity acquired after normalization in the method described above was used for hierarchical clustering. The clustering distance was calculated according to the coefficient related to different protein expression quantities.

**Fig. 6:**
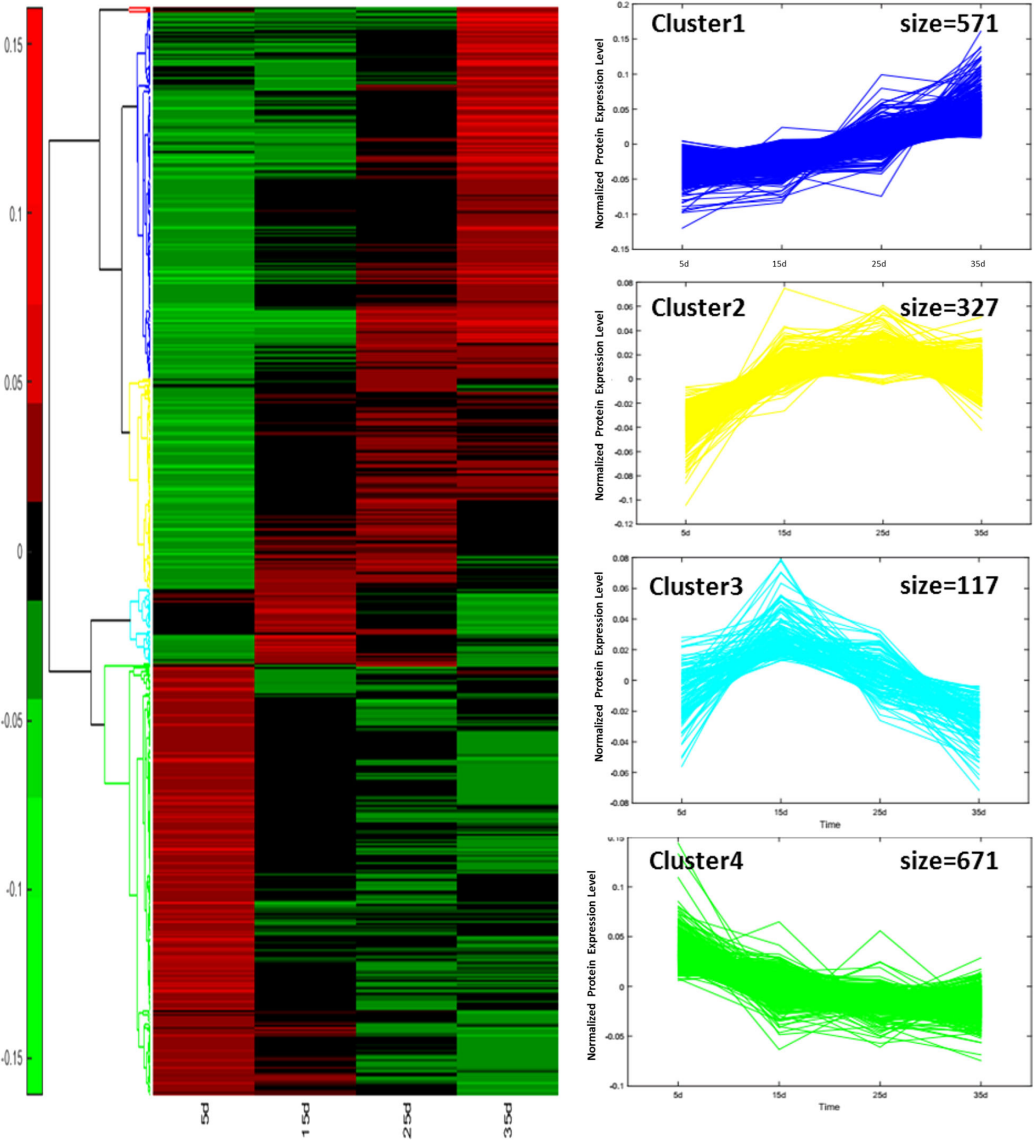
Hierarchical cluster analysis of proteins in different maturity periods. Four major clusters of protein changes were formed: Cluster 1 (*blue*)shows the proteins that were up-regulated in the whole growth period of fruit and reached a peak at 35DAA; Cluster 2 (*yellow*) reveals the proteins that were up-regulated and down regulated during the development of the fruit and reached a peak at 25DAA; Cluster 3 (*turquoise*) demonstrates the proteins that were up-regulated and down regulated during the development of the fruit and reached a peak at 15DAA; Cluster 4 (*green*) presents the proteins that were down-regulated in the whole growth period of fruit and reached a peak at 5DAA. Increasing intensities of red or green color indicate differentially up- or down-regulated proteins

Figure 6 illustrates the profile of 1694 proteins present during fruit ripening. Based on their relative abundance, 4 clusters of proteins were identified. The broken line graph of each category of protein is expressed in 4 colours: blue, yellow, turquoise and green. Overall, Cluster 1 (blue category) included 571 proteins that increased throughout the whole development stage and reached its peak value at 35 DAA. In Cluster 2 (yellow category), 327 proteins were identified that increased first and then decreased before reaching their peak value at 25 DAA. For Cluster 3 (turquoise category), 117 proteins were identified and reached their peak value at 15 DAA. Cluster 4 (green category) showed 671 proteins that were decreased in the development stage and reached their peak value at 5 DAA.

### Functional enrichment analysis of differential proteins

**Fig. 7:**
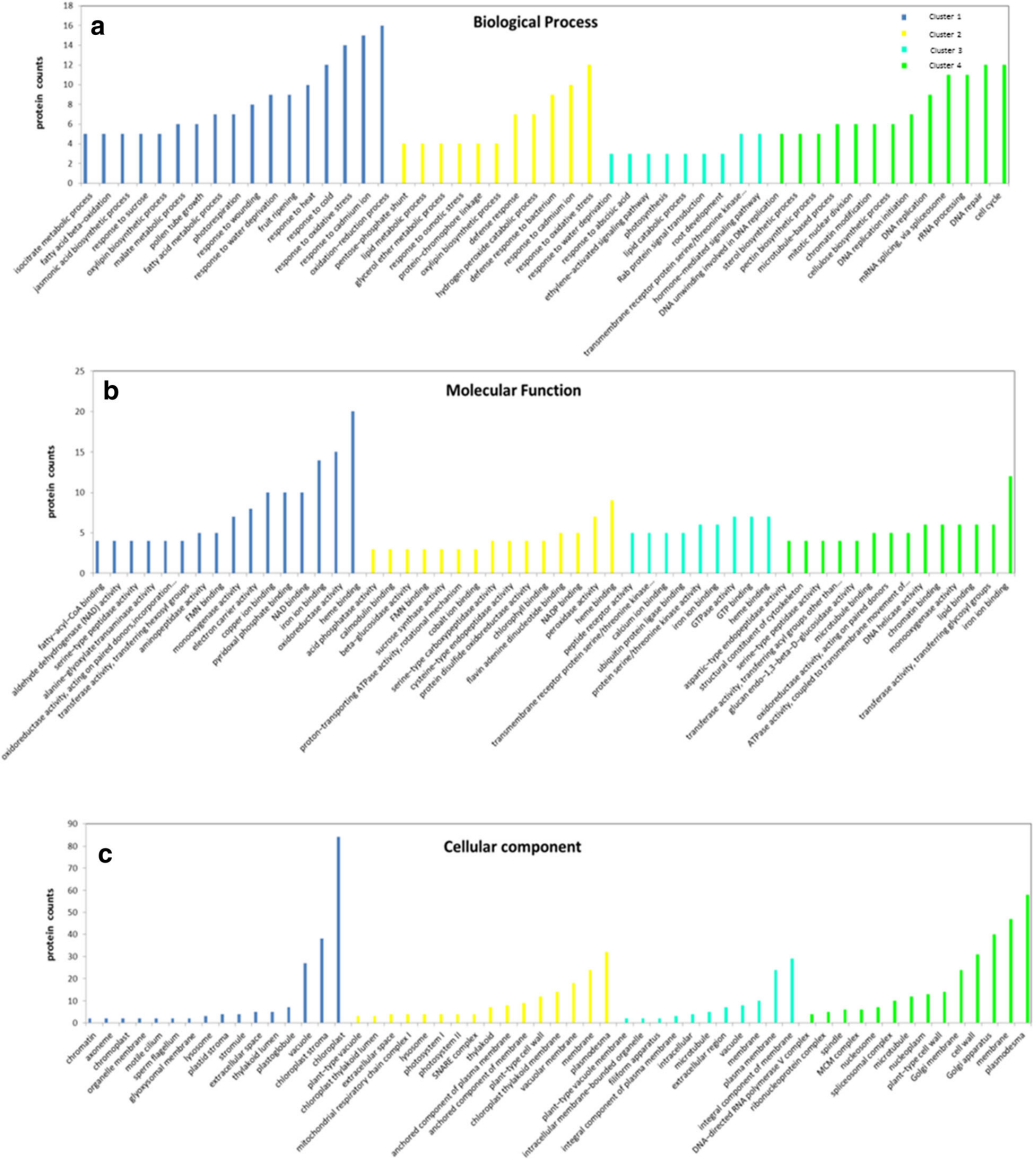
Enriched GO terms. **a** Biological process, **b** Molecular function and **c** Cellular component for the sequences annotated in the proteins in the four major clusters of protein. Color code for the four major clusters: Cluster 1 (*blue*), Cluster 2 (*yellow*), Cluster 3 (*turquoise*), Cluster 4 (*green*)

To further clarify the functions of these 4 categories of proteins, differentially expressed proteins were analysed using Gene Ontology (GO) function enrichment analysis. Among the 1694 changed proteins present in 4 clusters, their biological processes, molecular functions and cellular components were classified (Fig. 7).

The majority of up-regulated proteins in Cluster 1 (Fig. 6) had metal ion binding, pyridoxal phosphate binding, iron ion binding, NAD binding and oxidoreductase activities, which are involved in oxidation − reduction, fruit ripening, the response to oxidative stress, and the response to cold and heat processing. Those proteins are located in the chloroplast, vacuole, plastoglobule and thylakoid lumen (Fig. 7).

While many proteins were up-regulated first and then down-regulated in Cluster 2 (Fig. 6), these proteins had significant functions in heme binding, peroxidase activity, NADP binding, flavin adenine dinucleotide binding, chlorophyll binding and protein disulphide oxidoreductase activity as well as participating in the response to oxidative stress, hydrogen peroxide catabolic, oxylipin biosynthesis, protein − chromophore linkage, response to osmotic stress and glycerol ether metabolic processes, which are located mainly in the plasmodesmata, membrane and vacuole membrane (Fig. 7).

The majority of proteins in Cluster 3 (Fig. 6) have heme binding, GTP binding, GTPase and protein serine/threonine kinase activities and are involved in a hormone − mediated signalling pathway, a transmembrane receptor protein serine/threonine kinase signalling pathway and the root development process. Those proteins are located in the membrane and plasma membrane (Fig. 7).

While many down-regulated proteins in Cluster 4 (Fig. 6) showed significant functions in iron ion binding, transferase, lipid binding and monooxygenase activities, and transferring glycosyl groups, these proteins also participate in DNA repair and the mRNA splicing process. These proteins are located mainly in the plasmodesmata, membrane and Golgi apparatus (Fig. 7).

### Gene expression

**Fig. 8:**
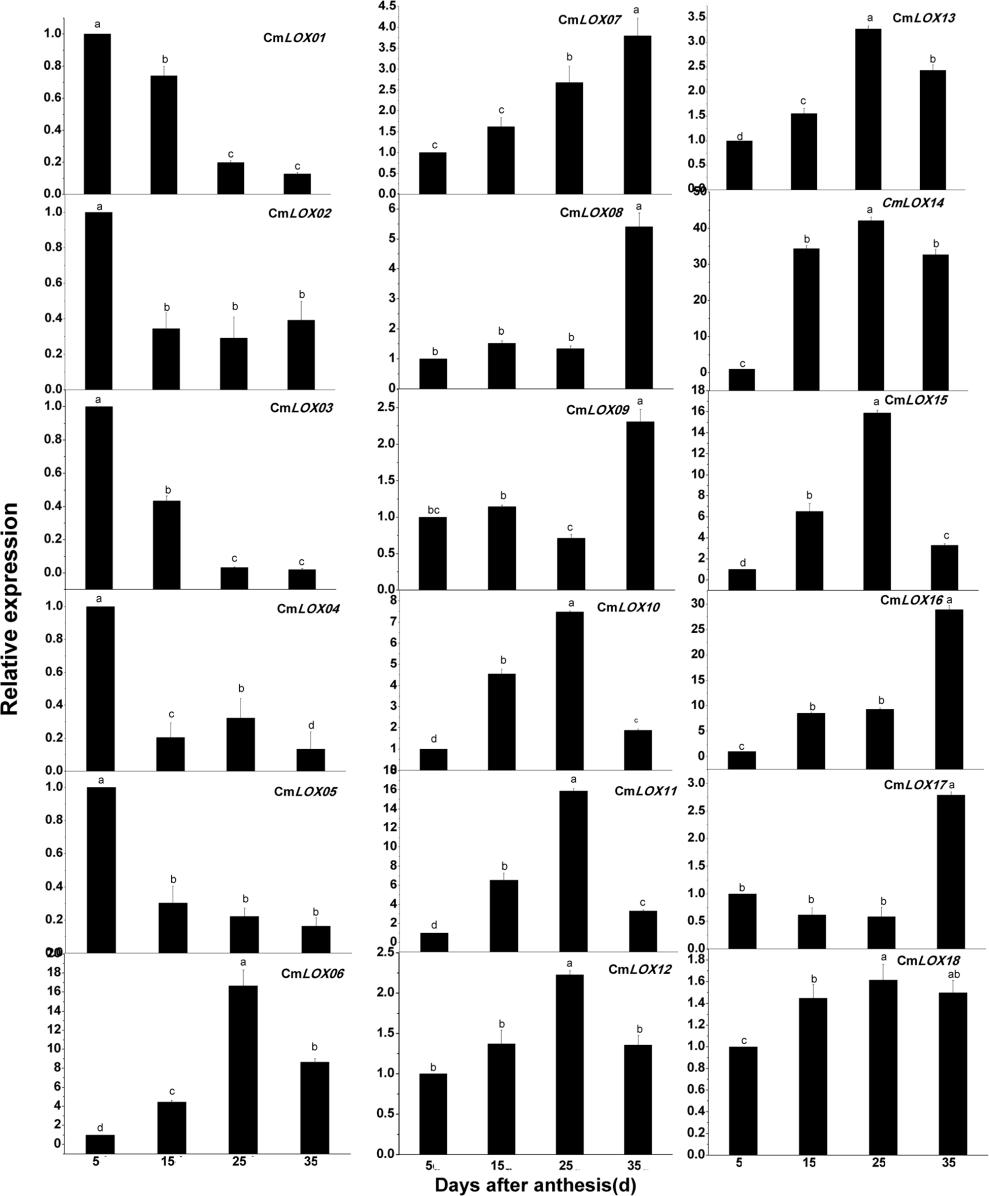
Expression analysis of 18 melon LOX genes in different maturity periods using Real-time RT-PCR. Real-time RT-PCR was performed using the primers specific to the 18 CmLOX genes. The expression level of CmLOXs in melon fruit at 5DAA was set as“1.0”. 18S primers giving a 148-bp product was as inner standard for each gene. The four maturity periods of melon included 5DAA, 15DAA, 25DAA and 35DAA

**Fig. 9:**
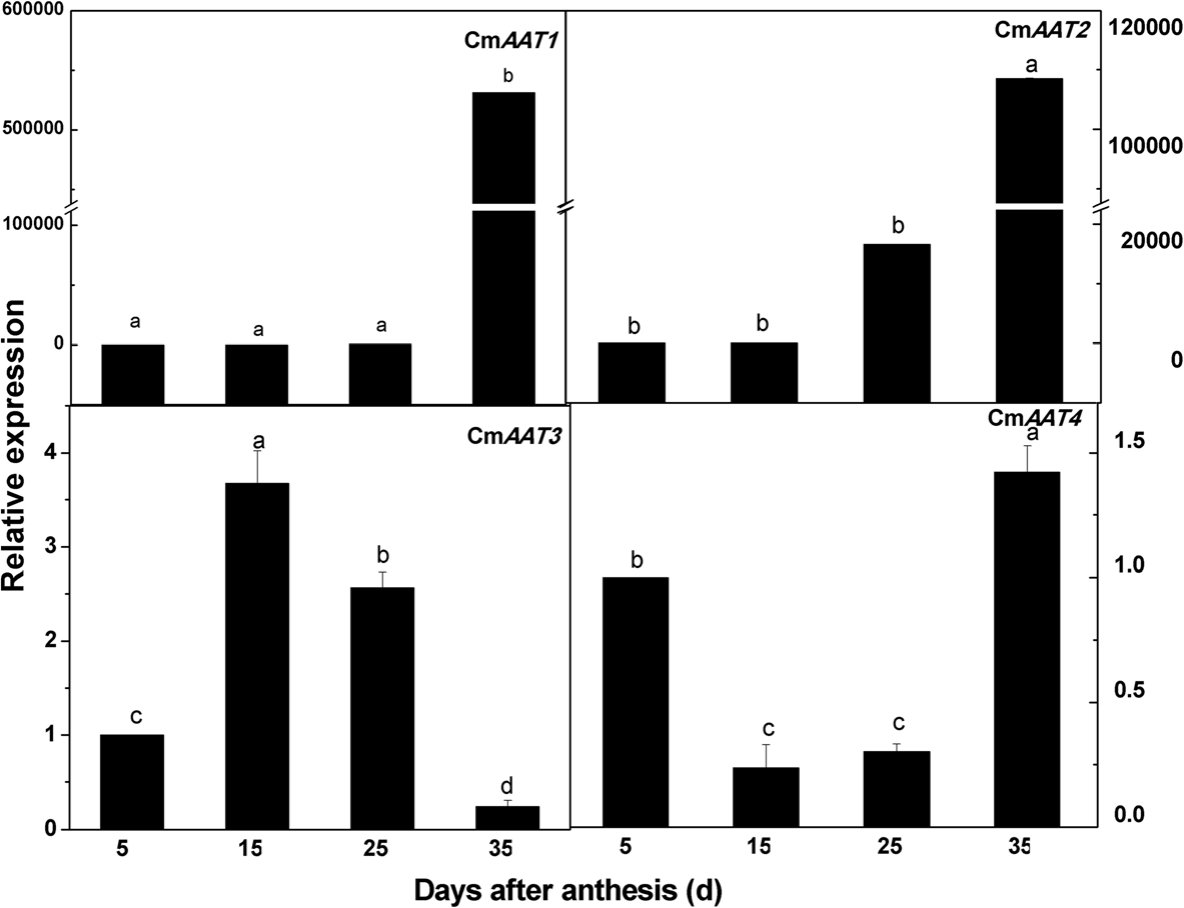
Expression analysis of 4 melon AAT genes in different maturity periods using Real-time RT-PCR. Real-time RT-PCR was performed using the primers specific to the 4 AAT genes. The expression level of CmAATs in melon fruit at 5DAA was set as“1.0”. 18S primers giving a 148-bp product was as inner standard for each gene. The four maturity periods of melon included 5DAA, 15DAA, 25DAA and 35DAA

Eighteen LOX genes, four AAT genes, an SS gene and an SPS gene in the melon genome were selected for transcriptional analysis to determine if gene expression data would confirm the changes in protein abundance. Expression analysis using real-time PCR showed that 18 CmLOXs and 4 CmAATs were constitutively expressed but varied greatly in the different ripening stages. The expression patterns of Cm*LOX01-05* showed preferential expression in the young stage at 5 DAA. While gene expression of Cm*LOX07-09*, Cm*LOX16* and Cm*LOX17* increased continuously in the ripening process and peaked at 35 DAA. Gene expression of Cm*LOX06*, CmLOX*10-15* and Cm*LOX18* increased at first, but then decreased after 25 DAA (Fig. 8). Except for Cm*AAT3*, the expression of Cm*AAT1*, Cm*AAT2* and Cm*AAT4* had a continuous synchronized increase and peaked at 35 DAA (Fig. 9). Cm*AAT1* and Cm*AAT2* were strongly expressed during melon ripening.

**Fig. 10:**
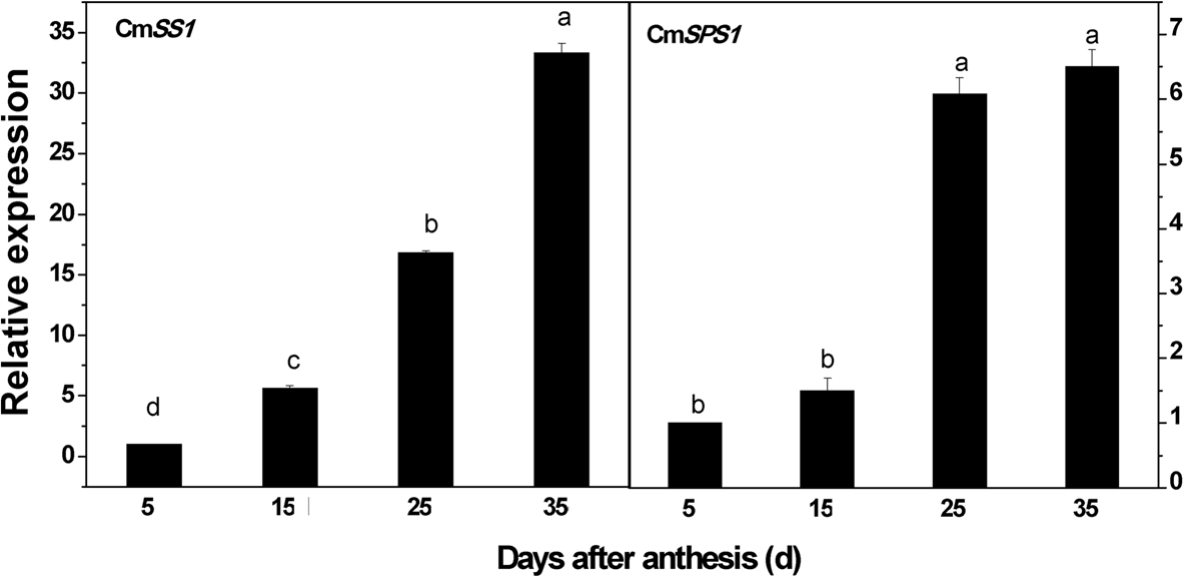
Expression analysis of CmSS1 and CmSPS1 genes in different maturity periods using Real-time RT-PCR. The expression level of CmSS1 and CmSPS1 in melon fruit at 5DAA was set as“1.0”. 18S primers giving a 148-bp product was as inner standard for each gene. The four maturity periods of melon included 5DAA, 15DAA, 25DAA and 35DAA

The pattern of changes at the transcript level was similar for Cm*SS1* and Cm*SPS1* from 5 DAA to 35 DAA with a gradual or sharp increase after 5 DAA (Fig. 10).

## Discussion

During the ripening of oriental melon, physiological changes lead to significant changes in the fruit that affect firmness, rind colour, SSC, soluble sugar content, ethylene content and aroma volatiles, which all affect fruit quality. In this study, we applied iTRAQ to identify significant changes in proteins that may be related to fruit metabolism during ripening and to obtain a global view of oriental melon ripening from a proteomic standpoint. The identified proteins were linked to metabolic processes, such as α-linolenic acid, glycolysis and starch and sucrose metabolism. The discussion that follows is based on those metabolic processes and the expression patterns of genes.

### α-Linolenic acid metabolism

**Fig. 11:**
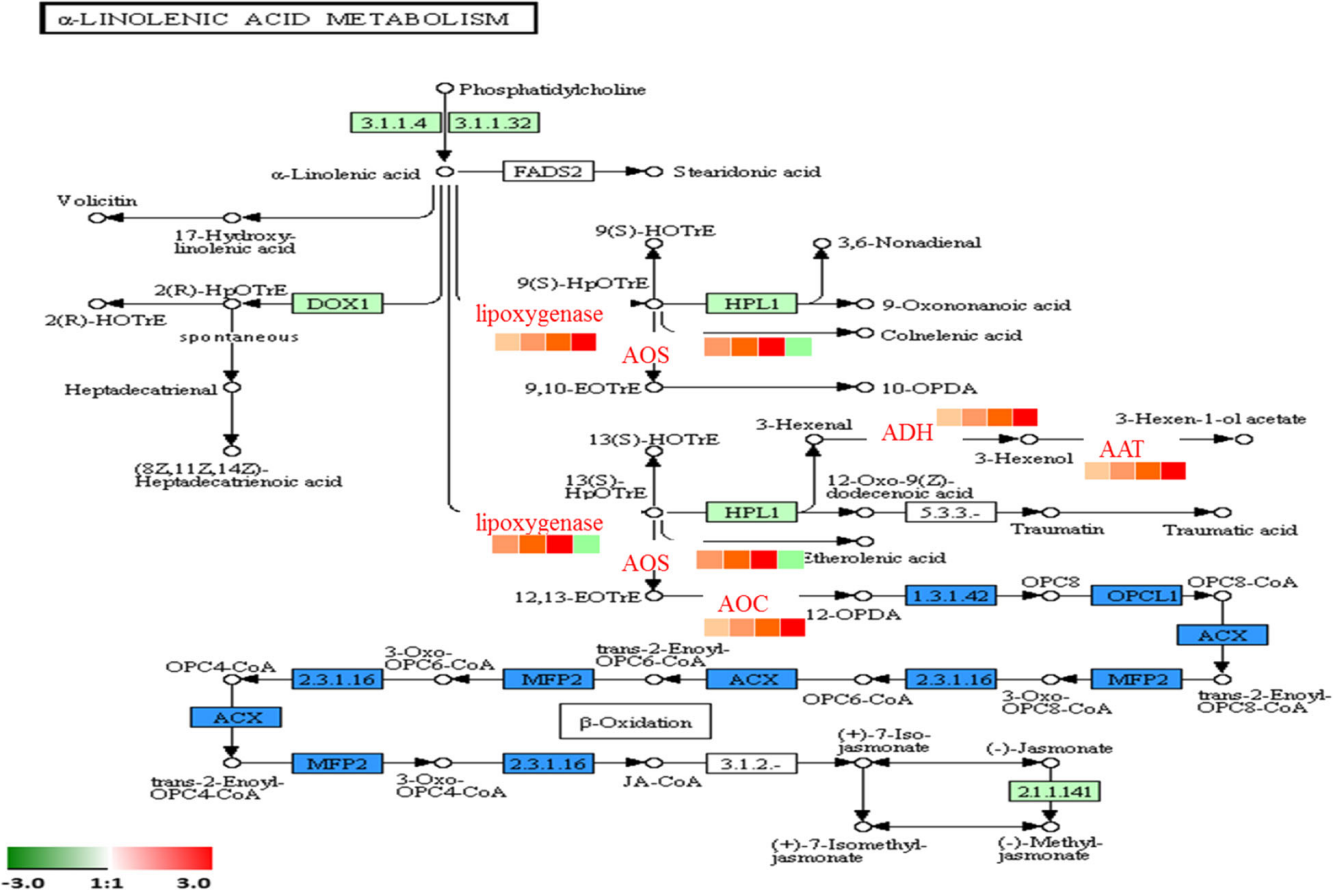
α-Linolenic acid metabolism. The protein levels of the regulated enzymes are shown in coloured squares. In sequence order (*left to right*), stages are displayed from 5DAA, 15DAA, 25DAA and 35DAA

α-Linolenic acid is the main substrate of fatty acid oxidation that is involved in the synthesis of alcohols, aldehydes, acids and esters in oriental melon volatile compounds. We found that total alcohol and ester contents increased significantly in ripening fruit. Total aldehydes showed a constant level throughout ripening, while total acids decreased significantly at 25 DAA (Fig. 2 ‘A’). In most oriental melon cultivars, the content of esters increased significantly during fruit development and ripening with an increase in major alcohols in mature fruit [1, 39], which agreed with our analysis. At the proteomic level, we identified and quantified several key enzymes related to aroma volatile biosynthesis, including alcohol dehydrogenase (ADH) and alcohol acetyltransferase (AAT). These were important enzymes to catalyse the formation of alcohols and esters, and these enzymes increased in oriental melon during ripening (Fig. 11). These results indicated that the aroma volatiles biosynthesis pathway was up-regulated from 5 DAA to 35 DAA, and the process for aldehydes to generate alcohols and esters mainly occurred at 35 DAA during fruit ripening. At the same time, many previous studies indicated that plant ADHs have been involved in seedling and pollen development as well as fruit development [40, 41]. For example, ADH was markedly increased at the pink and red fruit stages in ripening Chinese bayberry [27] and tomato [42] fruits. In contrast, the ADH protein decreased significantly in strawberry fruits at different ripening stages [43], which was not the same as our results. Therefore, we speculated that ADH expression may be related to sample type or another factor, which would be worthy of further study. However, the AAT protein showed similar expression patterns in many fruit during different developmental stages. For example, two AAT proteins (AAT1 and AAT2) increased more between the pink to red stages than from the white to pink stages in both cultivars of strawberry [22]. In cherimoya, an increase in AAT protein was observed during ripening [44]. Therefore, we speculated that AAT protein was prominently expressed later in fruit development. We also identified lipoxygenase (LOX) in different ripening stages, which catalysed the formation of 9(s)-HOTrE and 13(s)-HOTrE through 9-LOX and 13-LOX. At the proteomic level, the content of 9-LOX and 13-LOX peaked at 35 DAA and 25 DAA, respectively, during fruit ripening.

Characterization of the pathways at the α-Linolenic acid metabolism transcript level showed the differential expression pattern of LOX (Cm*LOX01-18*), ADH (Cm*ADH01-12*) and AAT (Cm*AAT1-4*).

In the present study, RT-qPCR analysis indicated that the expression patterns of 5 LOX genes, including *CmLOX01-05*, showed a relatively high abundance in the earlier stages of melon development, but their levels declined as the fruit developed (Fig. 8), which was similar to a previous study in oriental melons [45]. Previous studies showed a higher requirement for LOX activity for cell division and fruit growth during early fruit development. In grapes, the Vv*LOXC* transcript abundance steadily declined during the initial stage of berry growth [46]. In kiwi fruit, most of the LOX genes showed decreased levels during the late stages of fruit development [47]. These results suggest that these genes may play a role in early-stage fruit development.

Nevertheless, Cm*LOX06*, CmLOX*10-15* and Cm*LOX18* peaked at 25 DAA, which was similar to the protein expression pattern of 13-LOX. These genes were proposed to have 13-LOX activity and have been suggested to take part in the conversion of lipid hydroperoxides to jasmonic acid cyclic precursors and volatile compounds, such as aldehydes and alcohols [45]. Whereas, *CmLOX07-09*, *CmLOX16* and *CmLOX17* increased continuously in the ripening process and peaked at 35 DAA, which was similar to the protein expression of 9-LOX. Additionally, only *CmLOX07* and *CmLOX09* belonged to 9-LOX [45]. The results revealed that 9-LOX may play a role in organ development and fruit softening [48]. A previous study observed the expression of Tom*LOXB* and Tom*LOXC* in tomatoes, Md*Lox1* and Md*Lox7* in apples, and Ci*LOX* in watermelons during fruit development and ripening [49, 50]. Taken together, the 5 genes might be involved in fruit development and play a regulatory role in fruit ripening.

In our study, except for Cm*AAT3*, the expression levels of Cm*AAT1*, Cm*AAT2* and Cm*AAT4* were synchronized with the quantity change in proteins (Fig. 11). In apricot, Pa*-AAT* expression levels showed a sharp increase in the late-harvest stages [51]. In apple, the expression levels of Md*AAT1* and Md*AAT2* were increased as ripening progressed and were consistent with the total amount of esters detected between two cultivars [52]. Moreover, Cm*AAT1* and Cm*AAT2* were specifically expressed in fruits at increasing rates in the early and mid-phases in the ripening of melons. In addition, these genes are correlated with the total detected emission levels of volatile esters [53], which was the same as our results.

Manrı´quez et al. reported that Cm*ADH01* and Cm*ADH02* were both expressed specifically in fruit and up-regulated during ripening, which suggested that the ADH protein played a specific role in the regulation of aromatic biosynthesis in melons. The results at the transcript level reported by Jin et al. showed that, except for Cm*ADH01*, Cm*ADH09* and Cm*ADH11*, the expression quantity of all other genes peaked at 35 DAA [54], and this pattern was similar to the expression pattern for ADH protein. Therefore, this result suggested that many CmADHs may participate in fruit development during the developmental period for melons. Furthermore, in olea europaea, the Oe*ADH* transcript gradually increased along with olea fruit development and peaked at fruit ripening, and the change in Oe*ADH* expression was consistent with the olea fruit growth curve [55]. A similar pattern of ADH gene expression was found in apricot fruits [51].

The expression patterns of 64.7% of the genes were consistent with the expression patterns of their corresponding proteins. A discrepancy between the transcript and proteomic levels may indicate the presence of a certain regulatory mechanism in the initial stage of development in oriental melon that can influence the expression of corresponding proteins at the translation level.

Another branch of α-Linolenic acid metabolism is the formation of jasmonic acids through allene oxide synthase (AOS) and allene oxide cyclase (AOC). We also found that AOS and AOC reached their maximum expression at 25 DAA and 35 DAA, respectively. It was demonstrated that AOS was down-regulated and decreased in developing peach fruit [56]. Two JA-related genes (*AOS*) showed a low level of expression, and their transcript amounts initially decreased and then increased in the ripening process of peaches [57]. The discrepancy between our work and published results may indicate the presence of multiple isozymes of AOS in oriental melon fruit that are regulated differently in other species by both genetic and environmental factors. In oriental melon, We identified two isozymes of AOS in different ripening stages, which catalysed the formation of 10-OPDA and 12-OPDA and reached their maximum expression at 25 DAA.

### Starch and sucrose metabolism

**Fig. 12:**
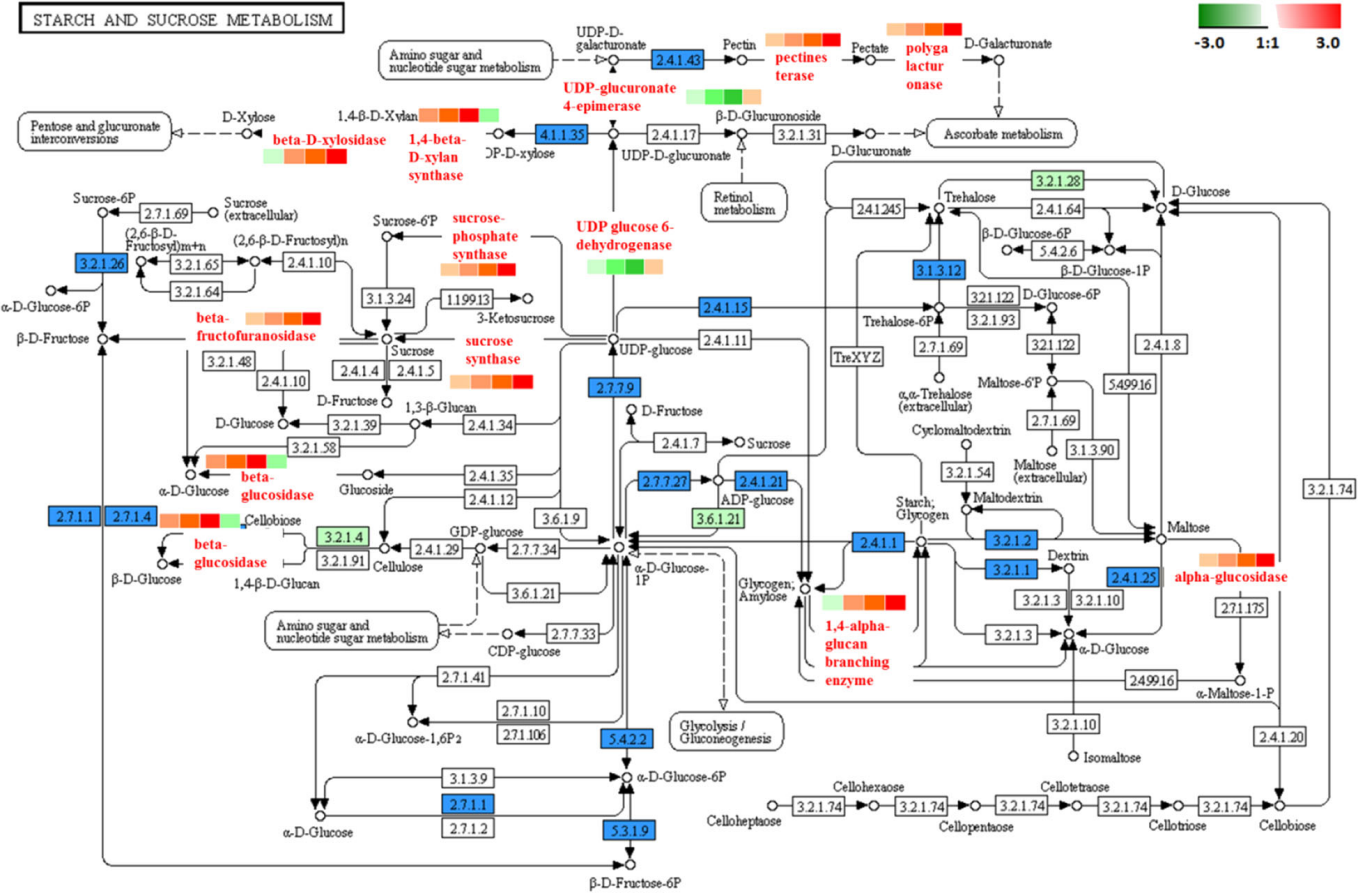
Starch and sucrose metabolism. The protein levels of the regulated enzymes are shown in coloured squares. In sequence order (*left to right*), stages are displayed from 5DAA,15DAA, 25DAA and 35DAA

Most of the soluble sugars accumulated in fruit are sucrose, glucose and fructose. Sucrose not only determines the sweetness and flavour of fruit but is also a basic compound for synthesizing other significant quality components and flavour substances, such as aromatic substances and pigments [58–61]. Starch and sucrose metabolism-related enzymes play an important role during fruit ripening and senescence. Our study clearly illustrated that sugar content was significantly increased as fruit ripening advanced from 5 DAA to 35 DAA (Fig. 2 ‘C’). The increase in sucrose content was well-correlated with the protein profiles associated with starch and sucrose metabolism in oriental melon. We identified and quantified two pathways for sucrose synthesis through sucrose synthetase (SS) and sucrose phosphate synthetase (SPS) (Fig. 12). The role of SS in sucrose synthesis is well-known in many fruits [62, 63]. SS is one of the most important enzymes to catalyse the formation of sucrose in oriental melons during ripening [64]. At the proteomic level, we found SS increased significantly during fruit ripening and reached its maximum expression at 35 DAA. In addition, we also found significant up-regulation of SPS. SPS was closely related to sugar accumulation, the formation of fruit quality, ripening and senescence [65, 66]. In two tomato species, SPS matched the changes in sucrose content and led to rapid sucrose increase during fruit ripening [67].

Gene expression of Cm*SS1* and Cm*SPS1* increased significantly during ripening in this study (Fig. 10). Therefore, the expression of these genes was related to fruit ripening. The activity of SS and SPS in apples reflected sucrose synthesis and accumulation, and expression of both genes increased significantly in the maturity stages [67, 68]. The three SPS genes of kiwi fruit increased in response to treatment with exogenous ethylene and again during climacteric ripening [69]. Nevertheless, the mRNA expression level of the SPS gene increased gradually during strawberry fruit development, and SS decreased continually during the white or turning stages [70]. Both precursors of sucrose, were up-regulated at 8 DAP and down-regulated during another oriental melon cultivar ‘Gotgam’ fruit maturation [71]. Furthermore, the expression level of Cm*SS1* showed a decreased trend in the development of melon fruit [72] that was not the same as our results. The role of SS in sweet melons in the previous studies was ambiguous and even contradictory. Sucrose synthase could catalyse and decompose sucrose as a substrate for the cell wall and starch [73]. Therefore, we speculated that SS appeared to be important for sucrose synthesis in oriental melons but not for starch formation. Our work provides proteomic evidence that SS and SPS become more abundant as rapid sucrose synthesis occurs in ripening oriental melon fruit.

UDP-glucuronate-4-epimerase, UDP-glucose-6-dehydrogenase, pectinesterase (PE) and polygalacturonase (PG) have been recognized as key enzymes in generating and deconstructing pectin [74]. Using the fruit firmness tester, we found that firmness declined significantly after 25 DAA. The firmness of the fruit was closely related to soluble pectin content [75]. PE and PG, respectively, catalysed the formation of pectate and D-galacturonate and accelerated the softening of fruit [76, 77]. At the proteomic level, there was a significant increase in these proteins as fruit ripened from 5 DAA to 35 DAA, which coincided with a decrease in firmness. At the same time, many previous studies indicated that the firmness decreased as the PG activity gradually increased in tomatoes [78], peaches [79], kiwi fruit [80] and other softening fruits. PG protein has been separated and purified from mature fruits, such as tomatoes and peaches. In tomatoes, the PG protein has 3 kinds of isozymes called PG1, PG2a and PG2b3. They accumulated gradually in the process of fruit ripening and softening [81, 82] and exhibited similar trends as the iTRAQ results. Barrett.et al. found that PE activity gradually increased during cherry maturation, and “Bartlett” and “La France”fruits had similar characteristics [83]. Interestingly, previous studies on durian and kiwi fruit indicated that PE activity had a downward trend in the process of postharvest fruit softening, and specific enzymes were not involved in regulating fruit softening. Therefore, we speculated that PE activity appeared to be related to sample type or another factor, which would be worth further study.

Additionally, two enzymes (UDP-glucuronate-4-epimerase and UDP-glucose-6-dehydrogenase) involved in the biosynthesis of pectin were identified and both decreased first then increased slightly at 35 DAA (Fig. 12). These results indicated that pectin accumulated in the early ripening stage and decomposed gradually to decrease fruit firmness.

### Glycolysis metabolism

**Fig. 13:**
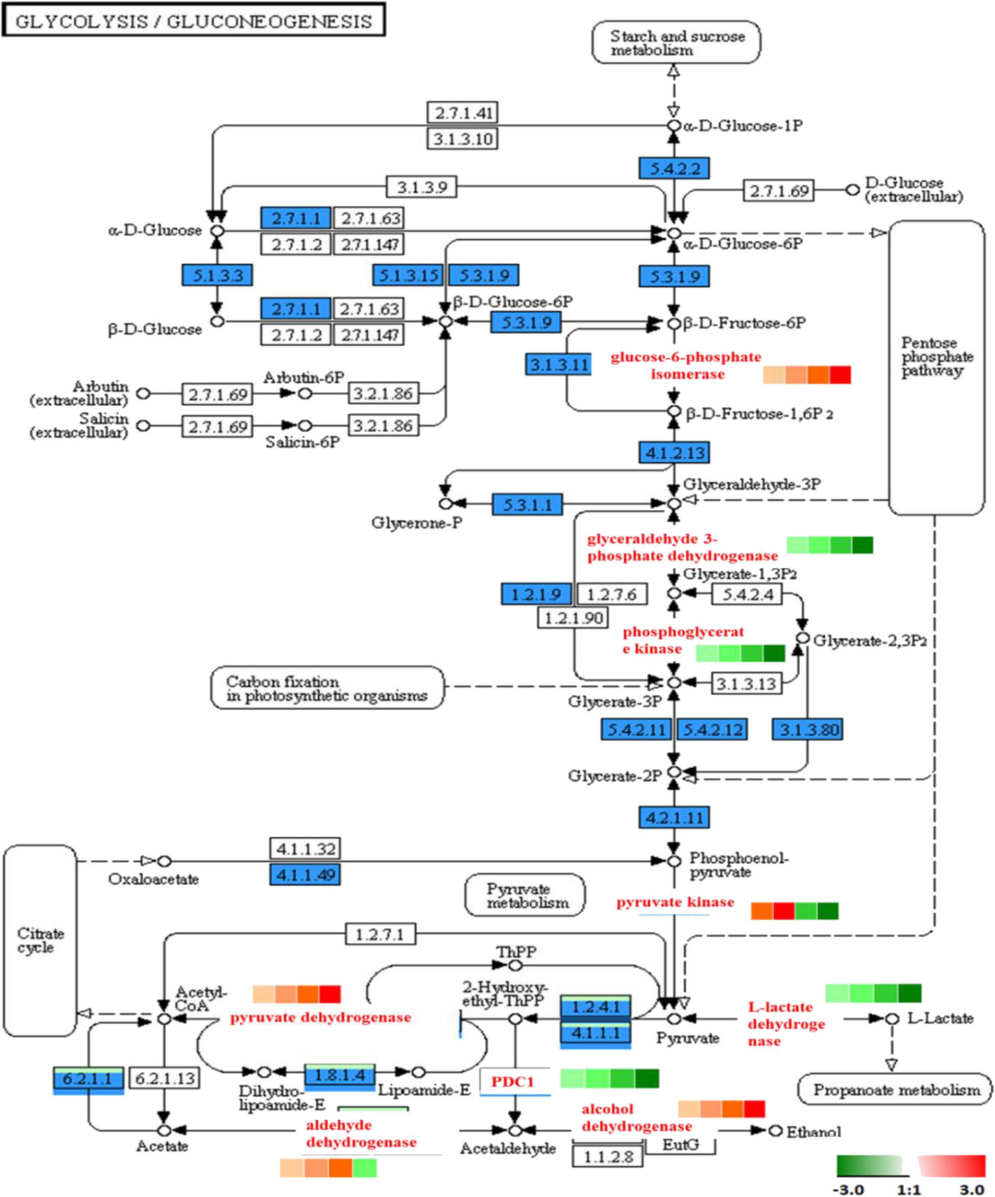
Glycolysis metabolism. The protein levels of the regulated enzymes are shown in coloured squares. In sequence order (*left to right*), stages are displayed from 5DAA, 15DAA, 25DAA and 35DAA

Glycolysis metabolism is a process in which the enzyme degrades glucose into pyruvic acid and generates ATP. It is a common metabolic pathway for glucose decomposition and generating energy in the cells of animals, plants and microbes [84]. In our study, several proteins involving glycolysis metabolism were identified. A phosphofructokinase (PFK), which converts fructose-6-phosphate (F6P) and ATP to fructose-1,6-diphosphate (FDP) and ADP, was identified and quantified as a protein in Cluster 1, and it increased significantly during ripening (Fig. 13). Interestingly, citric acid was recently reported to be involved in increasing ATP levels and to inhibit PFK activity [85]. It could be inferred that since acids decreased in the fruit maturation stage, the inhibiting effect on the enzyme was alleviated and its expression quantity increased.

In contrast, Phosphoglycerate kinase (PGK) and 3-phosphoglycerate dehydrogenase (GAPDH) decreased during fruit ripening and peaked at 5 DAA. Their combined action converted glyceraldehyde 3-phosphate (G3PDH) to glyceride 3-phosphate (G3P). GAPDH was recently reported to be involved in DNA replication, DNA repair, membrane fusion, RNA transport, microtubule fasciculation, and vesicular transport between the Golgi apparatus and endoplasmic reticulum [86]. This result was consistent with the GO enrichment of Cluster 4.

Pyruvate kinase (PK) catalyses phosphoenolpyruvic acid (PEP) to pyruvic acid (PA) and is an essential component for maintaining the glycolysis metabolic network. PK is mainly present in Cluster 3 and it reached its maximum expression at 15 DAA.

PA generated acetyl CoA through pyruvate dehydrogenase (PDH) under aerobic conditions. This process generally occurred during the maturation stage. Both the lactic acid generated through lactate dehydrogenase (LDH) under anaerobic conditions and the acetaldehyde generated under PDH conditions appeared in the immaturity stage and were significantly decreased during the maturation process.

We also identified aldehyde dehydrogenase (ALDH) as being initially up-regulated then down-regulated slightly during ripening. We further identified that ADH was up-regulated during fruit ripening. ALDH and ADH are responsible for generating acetate and ethanol respectively in fruit. At the proteomic level, they peaked at 25 DAA and 35 DAA respectively. Ethanol was up-regulated during watermelon and carambola fruit ripening [87, 88], while acetate was down-regulated at the later maturity of citrus [89], which agreed with our analysis. ALDH plays a significant role in fruit growth and development. Xiaoqin Li et al. [90] reported that the expression pattern of the ALDH gene superfamily in apples was similar to the results of the current study.

In this study, most differentially expressed proteins in the glycolysis metabolic pathway increased significantly during fruit ripening, and only a few enzymes decreased significantly. It could be speculated that most pathways of glycolysis metabolism were active during the maturation stage and only a few metabolic pathways were active during the immaturity stage.

## Conclusion

Oriental melon fruit showed significant changes in the production of firmness, rind colour, SSC, sugar content and volatile compounds during ripening. These physiological changes not only revealed the fruit’s ripening metabolism but provided an important link with dynamic protein changes obtained using iTRAQ which was the first time to study the protein profile in oriental melon fruits at different ripening stages.

These findings helped provide an understanding of metabolism pathways, such as glycolysis, α-Linolenic acid metabolism, and starch and sucrose metabolism in oriental melon fruit. All these processes largely determine final fruit quality. Some proteins underwent major changes in these pathways and could be used as new protein markers of oriental melon at different maturities. Consequently, this study may lead to new approaches for exploring the parameters controlling oriental melon development and ripening.

## Additional files

Additional file 1: Real-time PCR primers. (PDF 120 kb)
Additional file 2: Principal component loading matrix. (PDF 143 kb)
Additional file 3: Statistical results of protein identification. (PDF 90 kb)
Additional file 4: Distribution of the peptide fragment ions. (PDF 126 kb)
Additional file 5: Distribution of proteins relative molecular weight. (PDF 127 kb)
Additional file 6: Distribution of peptide sequence length. (PDF 130 kb)
Additional file 7: Distribution of peptides number. (PDF 126 kb)
Additional file 8: Correlation diagram of proteins. (PDF 294 kb)
Additional file 9: The proteins differentially expressed in four maturity stages of oriental melon. (XLSX 2405 kb)
Additional file 10: Hierarchical cluster analysis of proteins in different maturity periods. (PDF 259 kb)
Additional file 11: The raw data of firmness, rind colour, SSC, volatile compounds, suger content, ethylene production and expression analysis of genes. (XLSX 19 kb)

## Abbreviations

AAT: Alcohol acetyltransferase
ADH: Alcohol dehydrogenase
AGC: Automatic Gain Control
ALDH: Aldehyde dehydrogenase
AOC: Allene oxide cyclase
AOS: Allene oxide synthase
DAA: Days after anthesis
DIGE: Differencegel electrophoresis
EF: Error factor
FDP: Fructose-1, 6-diphosphate
FID: Flame ionization detector
F6P: Fructose-6-phosphate
GAPDH: 3-phosphoglycerate dehydrogenase
GC-MS: Chromatography-mass spectrometry
GO: Gene Ontology
G3P: Glyceride 3-phosphate
G3PDH: Glyceraldehyde 3-phosphate
HP: Headspace
iTRAQ: Isobaric tags for relative and absolute quantification
LDH: Lactate dehydrogenase
LOX: Lipoxygenase
PA: Pyruvic acid
PCA: Principal component analysis
PDH: Pyruvate dehydrogenase
PE: Pectinesterase
PEP: Phosphoenolpyruvic acid
PFK: Phosphofructokinase
PG: Polygalacturonase
PGK: Phosphoglycerate kinase
SPME: Solid phase micro extraction
SPS: Sucrose phosphate synthetase
SS: Sucrose synthetase
SSC: Soluble solids content
2-DE: Two-dimensionalgel electrophoresis
