# Supplementary material for: iTRAQ-based Protein Profiling and Fruit Quality Changes at Different Development Stages of Oriental Melon
Source: BMC Plant Biol. 2017 Jan 28;17:28. doi: 10.1186/s12870-017-0977-7 (PMC5273850; doi:10.1186/s12870-017-0977-7)
Supplement: Additional file 1: — Real-time PCR primers. (PDF 120 kb) [file 12870_2017_977_MOESM1_ESM.pdf]

Additional file 1. Real-time PCR primers

Gene	Primer	Sequence (5-3)
Cm*LOX01*	Cm *LOX01*-F	CCATCAACTTATCAGCCATT
	Cm *LOX01*-R	GTTCGTTCAAGAAGACCAT
Cm*LOX02*	Cm *LOX02*-F	TAGCACCGAAGGAATCAC
	Cm *LOX02*-R	AGACAGCACAATAACAGAGT
Cm*LOX03*	Cm *LOX03*-F	GACGACGAGAATGGAGAG
	Cm *LOX03*-R	GCTGGTTGAACTGTTGATAC
Cm*LOX04*	Cm *LOX04*-F	GCTCAGTGAAGTTATCAAGA
	Cm *LOX04*-R	GCTCAGTGAAGTTATCAAGA
Cm*LOX05*	Cm *LOX05*-F	GCTGCTTGTTCCTCATTA
	Cm *LOX05*-R	AGTCTTCAACTGCCATTC
Cm*LOX06*	Cm *LOX06*-F	GTGTATGTTCCAAGAGATG
	Cm *LOX06*-R	TGAATAAGTTGAGGAGTA
Cm*LOX07*	Cm *LOX07*-F	TACTTGGAGGAATGGATA
	Cm *LOX07*-R	TTGTTGTAACGGTGAGAC
Cm*LOX08*	Cm *LOX08*F	GGTAACTGGTCGTGGAAT
	Cm *LOX08*-R	AAGGCAGAGGAATAACAGAA
Cm*LOX09*	Cm *LOX09*-F	CAGATCCATCTTGTGAAC
	Cm *LOX09*-R	AGTTGGTAGAGTCATTCC
Cm*LOX10*	Cm *LOX10*-F	TGACAGGACAAGGAGTTC
	Cm *LOX10*-R	CGGTATTGGCAAGAATGTTA
Cm*LOX11*	Cm *LOX11*-F	CAAGTCATTCTCCAGATG
	Cm *LOX11*-R	GTTGATAAGGTCCAATCC
Cm*LOX12*	Cm *LOX12*-F	GTTAAGTTCTTCAGCATACG
	Cm *LOX12*-R	ACGAGGATGGATAGTAATG
Cm*LOX13*	Cm *LOX13*-F	CAAGCAACACAGGTAATG
	Cm *LOX13*-R	CTCCAGTTCTATTCTTCAAG
Cm*LOX14*	Cm *LOX14*-F	CAAGTGAACCAGATAACAAG
	Cm *LOX14*-R	CAGAGGAATTGGAATGAAG
Cm*LOX15*	Cm*LOX15*-F	CTATTATGCTGATGCTGAG
	Cm*LOX15*-R	GAAGGAAGTTGACAGATG
Cm*LOX16*	Cm *LOX16*-F	ATACGGACCTCAAGAATC
	Cm *LOX16*-R	GAGTCAAAGTGTCATCAG
Cm*LOX17*	Cm *LOX17*-F	TGACTATCTAATGCCACTTC
	Cm *LOX17*-R	CCAACTTATCTCTTCTCCT
Cm*LOX18*	Cm *LOX18*-F	TGGAGACTATCACAATCG
	Cm *LOX18*-R	CTTTCCCATCACCTCTAA
Cm*AAT1*	Cm *AAT1*-F	CCACAGGGGCCAGAATTACA
	Cm *AAT1*-R	TGGAGGAGGCAAGCATAGACTT
Cm*AAT2*	Cm *AAT2*-F	CTATAATTGGAGGGTGTGGAATTATC
	Cm *AAT2*-R	AACATTTGCCCTAAATCTTTCCAT
Cm*AAT3*	Cm *AAT3*-F	CGCTTGATGACATGGCACAT

	Cm *AAT3*R	GGCCTTACGGATAGCAGAGATC
Cm*AAT4*	Cm *AAT4*-F	CAGTTGTACCCCCGTCGAGTA
	Cm *AAT4*-R	AATATCGCTTCTGATCGGAACAC
Cm*SS1*	Cm*SS1*-F	ATTGCCTACCTTTGCCACT
	Cm*SS1*-R	TCCAGAATCCGTAAACTCCA
Cm*SPS1*	Cm*SPS1-F*	CCATGAAAAGAATGGCAGCTG
	Cm*SPS1-R*	CGGAACTGTCATAGCAAAAGAG
18srRNA	18srRNA-F	AAACGGCTACCACATCCA
	18srRNA-R	CACCAGACTTGCCCTCCA
