# Supplementary material for: iTRAQ-based Protein Profiling and Fruit Quality Changes at Different Development Stages of Oriental Melon
Source: BMC Plant Biol. 2017 Jan 28;17:28. doi: 10.1186/s12870-017-0977-7 (PMC5273850; doi:10.1186/s12870-017-0977-7)
Supplement: Additional file 2: — Principal component loading matrix. (PDF 143 kb) [file 12870_2017_977_MOESM2_ESM.pdf]

Additional file 2. Principal component loading matrix

Number	Volatile component	Principal component	
		1	2
v1	Ethyl Acetate	0.994	-0.106
v2	Butyl 2-methyl acetate	0.994	-0.106
v3	Hexyl acetate	0.994	-0.106
v4	Allyl acetate	0.994	-0.105
v5	Butyl acetate	0.999	-0.028
v6	Phenethyl acetate	0.994	-0.106
v7	2,3-Butanedioldiacetate	0.994	-0.106
v8	Allyl 2-ethyl butyrate	0.994	-0.106
v9	Methyl cyclopropane acetate	0.994	-0.106
v10	Ally decyl oxalate	0.993	0.117
v11	Ally nonyl oxalate	0.994	-0.106
v12	Benzyl acetate	0.994	-0.106
v13	Cinnamyl acetate	0.994	-0.106
v14	Birch-Me	-0.512	0.858
v15	Ethyl butyrate	0.994	-0.106
v16	Ally octyl oxalate	0.997	-0.066
v17	(E)-2-hexenyl acetate	0.997	0.080
v18	1-Ocenol	0.285	0.803
v19	4-Methyl 1-pentenol	0.709	0.672
v20	1-Butanol	-0.225	0.925
v21	3-Hexenol	0.964	0.254
v22	TRANS-3-HEXEN-1-OL	0.925	0.363
v23	1-HEPTEN-4-OL	0.988	0.149
v24	2-Nonynol	-0.651	-0.624
v25	2-Pentenol	0.830	0.534
v26	1-Nonanol	0.708	0.673
v27	3-Buten-1-ol	-0.225	0.926
v28	CIS-4-DECEN-1-OL	0.994	-0.105
v29	2-Methyl 4-nonanol	-0.566	-0.434
v30	Methylglyoxal	0.522	-0.670
v31	Benzaldehyde	0.971	0.230
v32	(E,E)-2,4-nonadienal	-0.224	0.926
v33	3-Butanolal	-0.380	-0.664
v34	Acetic acid glacial	-0.736	0.663
v35	Hydroperoxide,pentyl	-0.501	.784
v36	Myristic acid	0.964	.127
v37	Isobutyric acid	-0.225	.926
v38	9-Octadecyne	0.994	-.106
v39	Heptacosane	0.415	.654
v40	Allyl methyl sulfide	1.000	-.005
