# Supplementary material for: iTRAQ-based Protein Profiling and Fruit Quality Changes at Different Development Stages of Oriental Melon
Source: BMC Plant Biol. 2017 Jan 28;17:28. doi: 10.1186/s12870-017-0977-7 (PMC5273850; doi:10.1186/s12870-017-0977-7)
Supplement: Additional file 3: — Statistical results of protein identification. (PDF 90 kb) [file 12870_2017_977_MOESM3_ESM.pdf]

Additional file 3. Statistical results of protein identification

Database	Total spectra	Spectra	Peptides	Unique peptides	Protein groups
Cucumis melo	551042	81304	65426	36297	5835
