# Supplementary figures and images for: iTRAQ-based Protein Profiling and Fruit Quality Changes at Different Development Stages of Oriental Melon
Source: BMC Plant Biol. 2017 Jan 28;17:28. doi: 10.1186/s12870-017-0977-7 (PMC5273850; doi:10.1186/s12870-017-0977-7)

**Additional file 4. Distribution of the peptide fragment ions.**

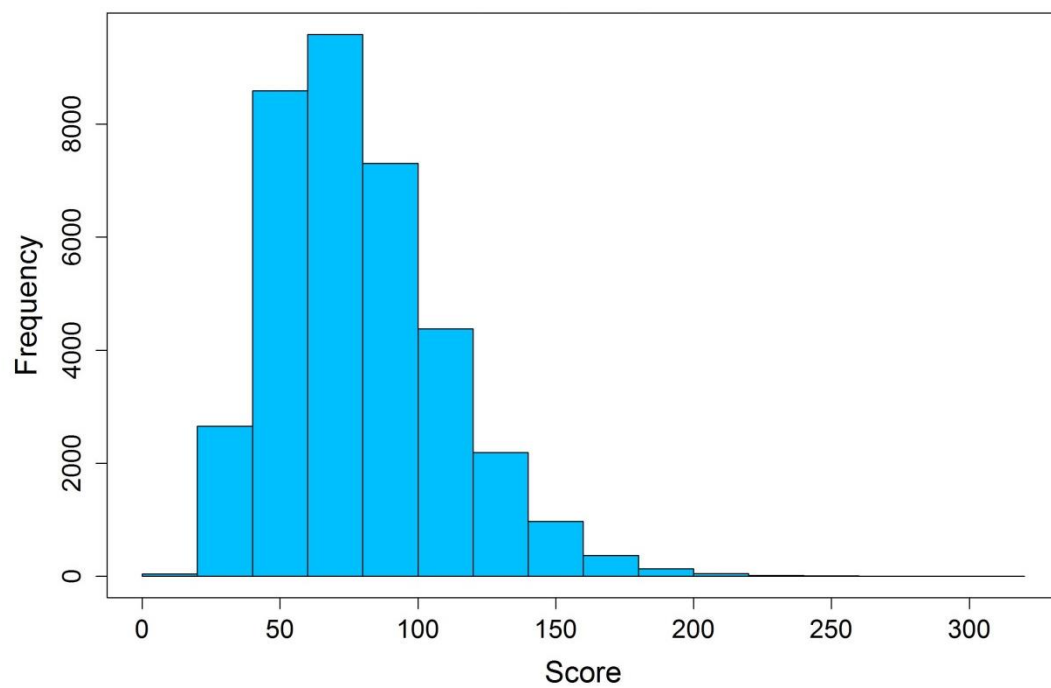

Supplement: Additional file 4: — Distribution of the peptide fragment ions. (PDF 126 kb) [file 12870_2017_977_MOESM4_ESM.pdf]

**Additional file 5. Distribution of proteins relative molecular weight**

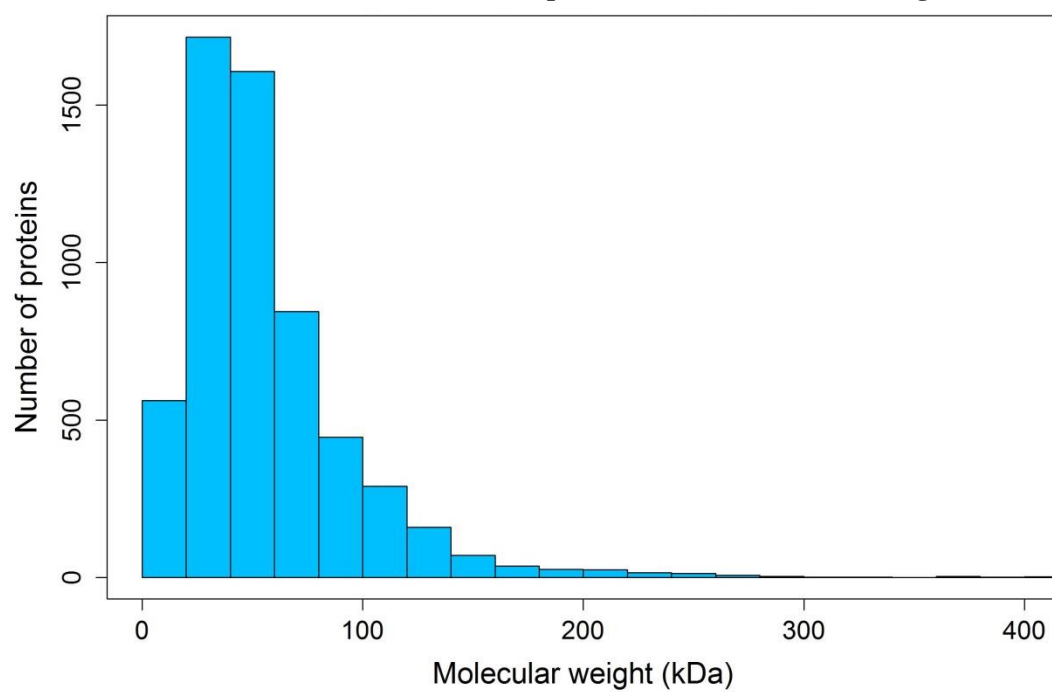

Supplement: Additional file 5: — Distribution of proteins relative molecular weight. (PDF 127 kb) [file 12870_2017_977_MOESM5_ESM.pdf]

**Additional file 6. Distribution of peptide sequence length**

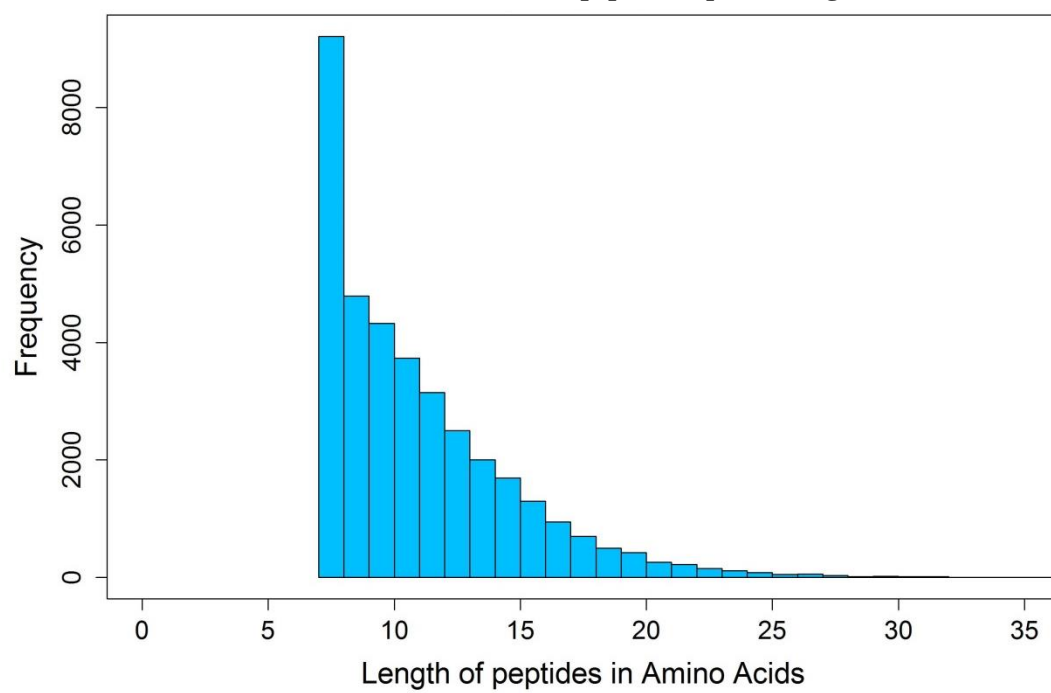

Supplement: Additional file 6: — Distribution of peptide sequence length. (PDF 130 kb) [file 12870_2017_977_MOESM6_ESM.pdf]

**Additional file 7. Distribution of peptides number**

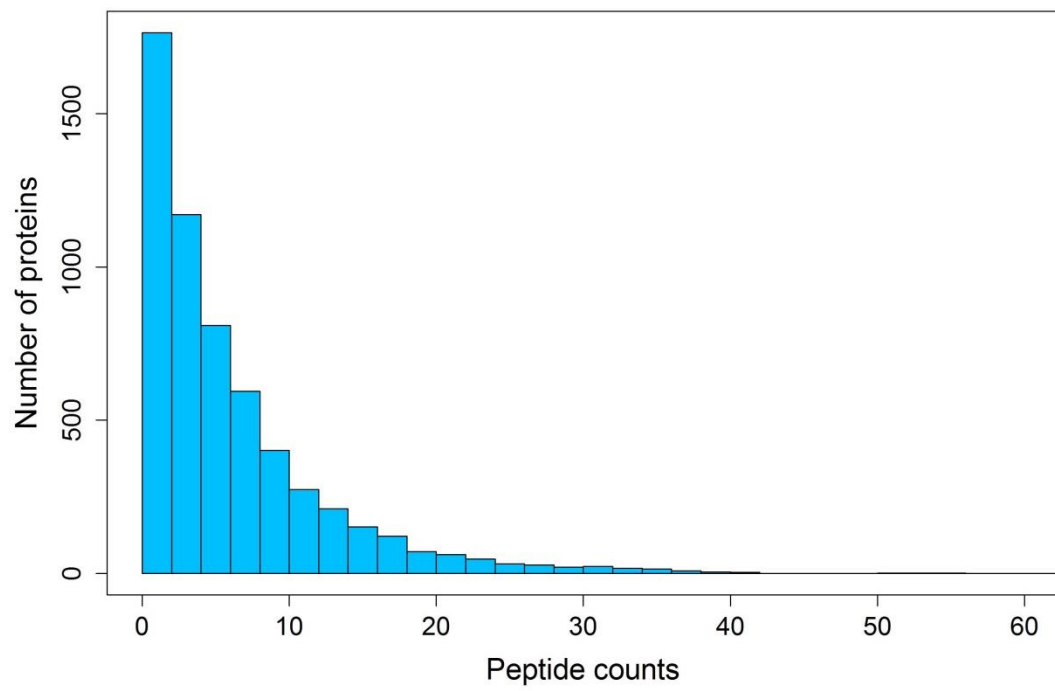

Supplement: Additional file 7: — Distribution of peptides number. (PDF 126 kb) [file 12870_2017_977_MOESM7_ESM.pdf]

Additional file 8. Correlation diagram of proteins

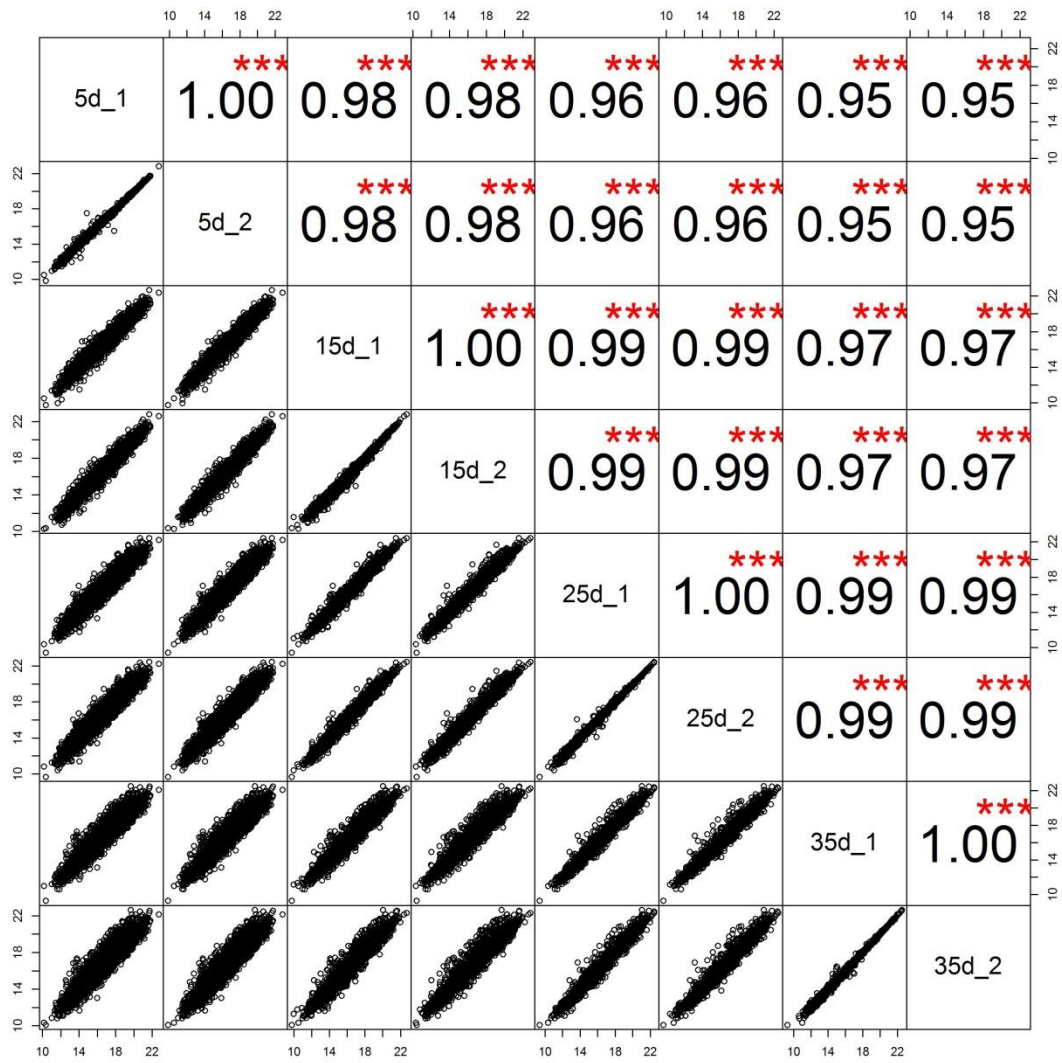

Supplement: Additional file 8: — Correlation diagram of proteins. (PDF 294 kb) [file 12870_2017_977_MOESM8_ESM.pdf]

Additional file 9. Hierarchical cluster analysis of proteins in different maturity periods.

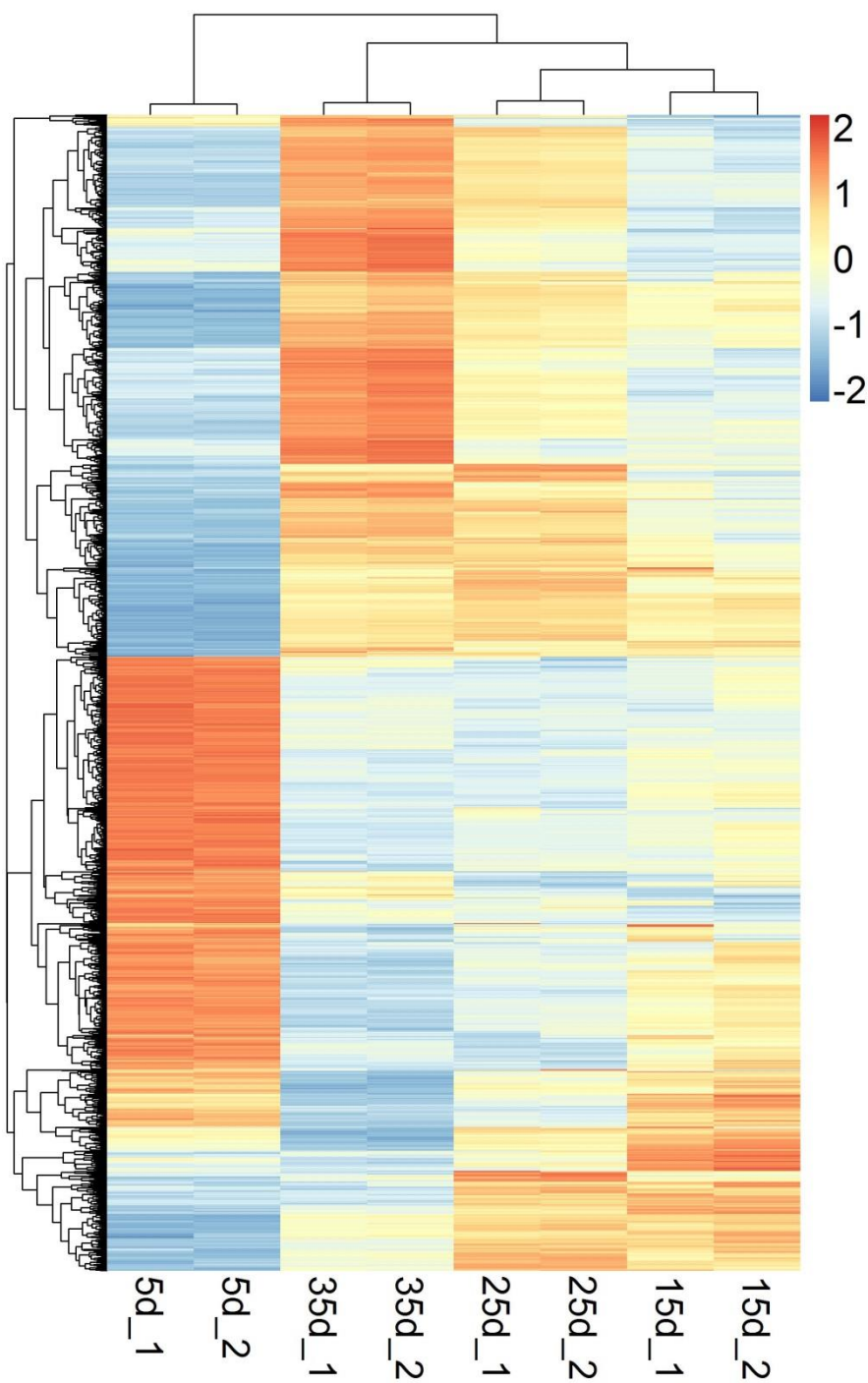

Supplement: Additional file 10: — Hierarchical cluster analysis of proteins in different maturity periods. (PDF 259 kb) [file 12870_2017_977_MOESM10_ESM.pdf]
